# Phylogeographic Patterns and Genetic Diversity of *Anopheles stephensi*: Implications for Global Malaria Transmission

**DOI:** 10.3390/tropicalmed10040109

**Published:** 2025-04-16

**Authors:** Jehangir Khan, Dongjing Zhang, Saber Gholizadeh, Yidong Deng, Abdul Aziz, Jianhuang Chen, Pir Tariq Shah, Zhiyue Lv, Tao Chen

**Affiliations:** 1 Hainan General Hospital, Hainan Affiliated Hospital of Hainan Medical University, Haikou 570100, China; 2 Laboratory of Tropical Veterinary Medicine and Vector Biology, School of Life Sciences, Hainan University, Haikou 570228, China; 3 Zhongshan School of Medicine, Sun Yat-sen University, Guangzhou 510080, China; 4 Zoology Department, Abdul Wali Khan University Mardan, Mardan 25000, Pakistan; 5 Cellular and Molecular Research Center, Cellular and Molecular Medicine Institute, Urmia University of Medical Sciences, Urmia 37100, Iran; 6 Medical Entomology and Vector Control Department, School of Public Health, Urmia University of Medical Sciences, Urmia 37100, Iran; 7 Nuclear Institute for Food and Agriculture, Peshawar 25000, Pakistan; 8 Faculty of Medicine, School of Basic Medical Sciences, Dalian University of Technology, Linggong Road, Dalian 116024, China; 9 Key Laboratory of Tropical Disease Control, Sun Yat-sen University, Ministry of Education, Guangzhou 510080, China; 10 Hainan Provincial Bureau of Disease Prevention and Control, Haikou 570100, China

**Keywords:** *Anopheles stephensi*, morphology, genetic diversity, population structure, urban malaria, Khyber Pakhtunkhwa

## Abstract

**Background**: *Anopheles stephensi*, a primary malaria vector in South Asia, is expanding its geographic range, raising concerns about increased malaria transmission. However, critical aspects of its genetic diversity, population structure, and evolutionary dynamics remain poorly understood in Khyber Pakhtunkhwa (KP), Pakistan, an endemic malaria region where *An. stephensi* is adapting to urban settings, posing challenges for the development of targeted vector control strategies. This study addresses this gap by analyzing COI, COII (cytochrome oxidase subunit I and II), and ITS2 (internal transcribed spacer 2) sequences from *An. stephensi* populations in KP and comparing them with global isolates. Additionally, egg morphology analysis was conducted to identify the biological form. **Methods**: Mosquitoes were collected from malaria-endemic districts (Nowshera, Charsadda, and Peshawar) using ovitraps. Eggs were characterized morphologically, and DNA was extracted for PCR amplification of COI, COII, and ITS2 markers. Sequences from 17 Pakistani isolates, along with global sequences, were analyzed. Phylogenetic relationships, haplotype networks, genetic diversity, and neutrality tests (Tajima’s D and Fu’s Fs) were assessed. **Results**: Egg morphology confirmed the mysorensis form (13–15 ridges per egg) in KP. COI sequences clustered into two subclades (Punjab and KP), with >99% similarity to global isolates. COII and ITS2 sequences showed high similarity (99.46–100%) with populations from China, Iran, India, and Brazil, reflecting strong genetic connectivity rather than distinct regional clustering. Haplotype analysis identified six COI, ten COII, and ten ITS2 haplotypes, with Hap_2 (50.7%) and Hap_1 (43.3%) being the most prevalent in COI, Hap_7 (29.4%) in COII, and Hap_3 (80.8%) in ITS2. Population genetic analysis revealed higher COI diversity in Pakistan and India, with moderate diversity in COII. Neutrality tests suggested balancing selection in COI for both countries, while COII and ITS2 indicated population contraction in Iran. **Conclusions**: The findings reveal strong genetic connectivity within regions (e.g., Pakistan) and differentiation across global populations of *An. stephensi*, highlighting its potential for further expansion and adaptation. These insights are critical for informing global malaria control strategies, particularly in regions vulnerable to vector invasion.

## 1. Introduction

Malaria remains a major global health burden, with an estimated 263 million cases and 597,000 deaths in 2023, an increase of 11 million cases from 2022 [1]. Despite control efforts, factors such as urbanization, climate change, and the vector expansion continue to impede elimination [2]. A key concern is the range expansion of *Anopheles stephensi*, an urban malaria vector spreading from South Asia and the Middle East into Africa [2,3,4].

*An. stephensi* demonstrates remarkable ecological adaptability, thriving in artificial breeding sites and urban environments, making it a persistent challenge for malaria control [5]. The species is classified into three biological forms: type, mysorensis, and intermediate, each differing in vectorial capacity and distribution [6,7,8,9,10]. The type form is an efficient urban vector, while mysorensis exhibits lower capacity in rural settings. The intermediate form, found in rural and peri-urban areas, has an uncertain vectorial role [8,9,11]. Identifying these forms is essential for targeted interventions. The type form requires urban-focused measures, such as larviciding in artificial containers, while mysorensis may necessitate rural habitat management. Egg ridge counts provide a key diagnostic trait for this purpose [8,12,13,14,15]. The genetic variability within *An. stephensi* influences its vector competence, insecticide resistance, and dispersal dynamics [16,17]. Recent reports from Africa highlight the species’ rapid adaptation to new environments, raising concerns about its potential to undermine malaria control in urban regions [18].

In Pakistan, malaria remains a pressing public health crisis, with *Plasmodium vivax* driving approximately 80% of cases, establishing it as the dominant species [3]. Once primarily a rural vector, *An. stephensi* has emerged as a significant contributor to malaria transmission in urban and peri-urban settings in the country [19,20,21]. This vector is widespread, dominating in urban centers like Karachi in Sindh Province and exhibiting a strong presence across Punjab’s rural and urban landscapes [19,21]. In Khyber Pakhtunkhwa (KP), a province burdened by high *P. vivax* prevalence, *An. stephensi* is increasingly prominent in urban areas such as Peshawar, yet its population dynamics and contribution to transmission remain insufficiently documented due to sparse surveillance data [19,20]. Despite its rising significance, the genetic diversity and population structure of *An. stephensi* in KP—key factors influencing its adaptability and vectorial capacity—are poorly understood, hampering the development of targeted control measures [19,20]. This knowledge gap underscores the critical need for genetic and phylogeographic insights to inform region-specific strategies, mitigating the growing threat of urban malaria across Pakistan and globally.

This study investigates the genetic diversity, phylogeography, and egg morphology of *An. stephensi* populations in KP using mitochondrial (COI and COII) and nuclear (ITS2) markers. We construct phylogenetic trees and phylogeographic networks to uncover patterns of genetic connectivity and differentiation. The specific objectives are to (i) characterize the *An. stephensi* biological form based on egg ridge counts and genetic markers, (ii) explore regional and global phylogeographic relationships, and (iii) infer the evolutionary dynamics underlying the species’ adaptability and spread. By integrating genetic and phylogeographic data, the current study will provide critical insights into *An. stephensi*’s population biology and support evidence-based strategies for mitigating its growing public health impact.

## 2. Materials and Methods

### 2.1. Study Area

**Figure 1:**
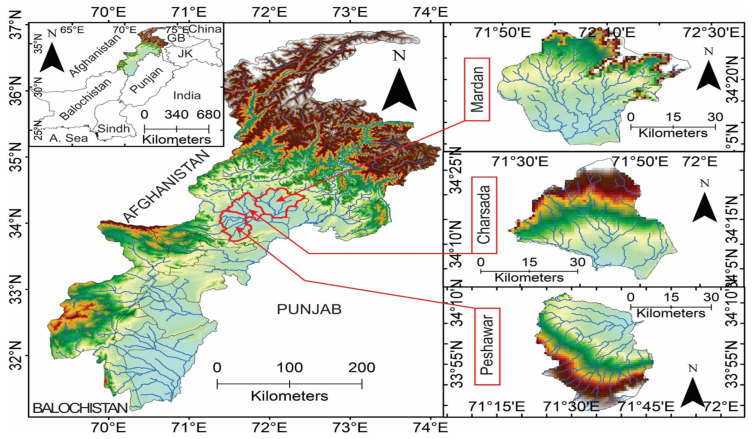
Map of Khyber Pakhtunkhwa highlighting the three districts selected for sampling.

Khyber Pakhtunkhwa (KP) Province, located in northwestern Pakistan (34.9526° N, 72.3311° E) (Figure 1), is the country’s fourth largest by area (101,741 km^2^) and third by population (35 million) [22]. It shares a western border with Afghanistan and is characterized by diverse geographical features, including the Hindu Kush mountains and major rivers such as the Indus and Kabul. This geographical diversity, coupled with a varied climate, presents unique challenges for malaria control by influencing vector distribution, breeding habitats, and transmission dynamics [23]. The climate in KP is marked by significant temperature fluctuations, ranging from 7 °C in January to over 40 °C during the peak summer months of May to July. Rainfall is also highly variable, with northern areas like Abbottabad receiving approximately 1532 mm annually, while southern areas such as Peshawar average around 817 mm [24]. The monsoon season (July–September) brings the majority of the rainfall, raising humidity levels and creating favorable conditions for mosquito proliferation and malaria transmission [24,25]. KP’s healthcare infrastructure comprises a network of hospitals and clinics alongside vector control interventions, including insecticide-treated nets (ITNs) and indoor residual spraying (IRS). However, resource limitations and accessibility challenges, particularly in rural areas, hinder effective malaria control efforts, exacerbating the province’s vulnerability to outbreaks. KP has a history of malaria outbreaks, with Peshawar serving as a key transmission hub due to its high population density, urban breeding sites, and reported malaria incidence, impacting neighboring districts like Charsadda and Mardan [26]. For example, Rowland et al. (1997) [26] documented sustained *Plasmodium vivax* transmission in Afghan refugee settlements near Peshawar, highlighting its role in regional malaria dynamics.

### 2.2. Mosquito Sampling and Colony Maintenance

Field sampling was conducted in three malaria-endemic districts (Figure 1) characterized by high malaria incidence and diverse ecology: Peshawar, Nowshera, and Charsadda. Sampling sites across these districts are detailed in Table 1. Collections occurred during the peak malaria season (September–December 2023), with 15–20 ovitraps placed at 100 m intervals in each area to ensure comprehensive coverage, following our previous study [27]. Each ovitrap consisted of a small, black cup filled to one-third capacity with water. A strip of egg-laying paper, designed to enhance mosquito oviposition, was placed inside each trap. These traps were checked twice weekly for egg/larvae collection. The collected samples were transported to the insectary at the Nuclear Institute for Food and Agriculture (NIFA), Tarnab, Peshawar, KP, where they were maintained at 28 ± 2 °C, 70 ± 5% RH, with a 12:12 (L) photoperiod, and provided with Khan Diet [15]. The resulting pupae were isolated and transferred to adult cages (30 × 30 × 30 cm) for emergence and provided with a 10% (*w*/*v*) sugar solution. The adult mosquitoes were then identified morphologically using previously published keys [28].

### 2.3. Morphological Study of Mosquito Egg

Five to six days after emergence, adult female mosquitoes were blood fed for 30 min on anesthetized mice. Following feeding, ovicups with water and filter papers were provided for oviposition. Eggs laid by mosquitoes collected from each district were analyzed morphometrically as described previously [15]. For each district, 100 eggs were placed on a slide with a drop of water and examined under a stereomicroscope at 40× magnification (bright field illumination) to count the ridges on one side, following the method reported by [14]. Additionally, scanning electron microscopy was used to capture detailed images of the egg structure [15].

### 2.4. DNA Extraction

Total DNA was extracted from each mosquito using the Qiagen DNeasy Blood and Tissue Kit (Qiagen, Hilden, Germany). Briefly, each individual female mosquito was placed in a 1.5 mL Eppendorf tube with 500 μL STE buffer and a sterile steel bead for enhanced cell lysis. The sample was homogenized (50 Hz for 30–60 s) using a tissue homogenizer according to our previous study [15]. DNA quality and quantity were assessed using a Nanodrop spectrometer (Thermo Scientific, Waltham, MA, USA), and concentration and purity were confirmed on 1% agarose gels. The extracted DNA was then stored at −20 °C or immediately processed for target gene amplification.

### 2.5. PCR Amplification and Sequencing

A total of 150 DNA samples, comprising 50 randomly selected samples from each district, were processed for PCR amplification of the partial COI gene using primers FP: 5′-TTGATTTTTTGGTCATCCAGAAGT-3′ and RP: 5′-TAGAGCTTAAATTCATTGCACTAATC-3′, the COII gene using primers FP: 5′-ATGGCAACATGAGCAAATT-3′ and RP: 5′-CCACCCTTTCTGAACATTGACC-3′, and the ITS2 region with primers FP: 5′-ATCACTCGGCTCGTGGATCG-3′ and RP: 5′-ATGCTTAAATTTAGGGGGTAGTC-3′. PCR reactions were carried out in a final volume of 25 μL. The PCR products were visualized on 1% agarose gel and purified using the Qiagen gel extraction kit (Qiagen, Hilden, Germany). The PCR thermal profiles for each primer are detailed in our previous article [15]. Finally, the amplified fragments were sent to a sequencing company in Guangzhou, China, for bidirectional Sanger sequencing.

### 2.6. Sequences and Phylogenetic Analysis

The sequences were processed to eliminate primer and nucleotide contaminants and were rechecked using Chromas software version 2.31 (www.technelysium.com.au/chromas.html, accessed on 15 January 2025). The final sequences were aligned with homologous sequences from GenBank using BLAST version 2.14.0 [29]. To ensure accurate phylogenetic inference, outgroup sequences from *An. gambiae*, *An. Funestus*, and *An. arabiensis* were included. All retrieved sequences, including outgroups, were included to root the trees and confirm monophyly of *An. stephensi*. All retrieved sequences, including outgroups, were aligned using ClustalW [30] for multiple sequence alignment and refined with BioEdit v7.2.5. Maximum likelihood (ML) phylogenetic trees [31] were generated using IQ-TREE (multicore version 1.6.12) under the best-fit substitution models TN + F for COI and K3Pu + F + G4 for COII, while K2P for ITS2 used the IQ tree model finder. Tree robustness was evaluated using 1000 bootstrap replicates and SH-like aLRT (Shimodaira–Hasegawa-like approximate likelihood ratio tests) with 1000 replicates, following the methodologies outlined by Nguyen et al. (2015) [32] and Hoang et al. (2018) [33]. The resulting phylogenetic trees were visualized and customized using FigTree v1.4. Moreover, PopArt [34] and DnaSP6 [35] were used to generate and analyze haplotype networks, inferring population dynamics and potential gene flow.

### 2.7. Phylogeographic Clustering and Population Genetics Analysis

The phylogeographic network analysis of population-specific genetic data provides a robust approach for mapping genetic connections among intraspecific sequences and visualizing relationships between populations and their frequencies [34]. To this end, we utilized the Minimum Spanning Network (MSN) tool available in PopArt v1.7 [34] to analyze our COI, COII, and ITS2 sequences of *An. stephensi*. To gain preliminary insights into the relationships between the identified haplotypes and global haplotypes, we conducted a phylogenetic analysis using COI, COII, and ITS2 sequences data retrieved from GenBank. Moreover, nucleotide diversity, the number of segregating sites, haplotypes diversity, and Tajima’s D statistic were calculated using PopArt v1.7 [34].

## 3. Results

### 3.1. Egg Morphometric Analysis

**Figure 2:**
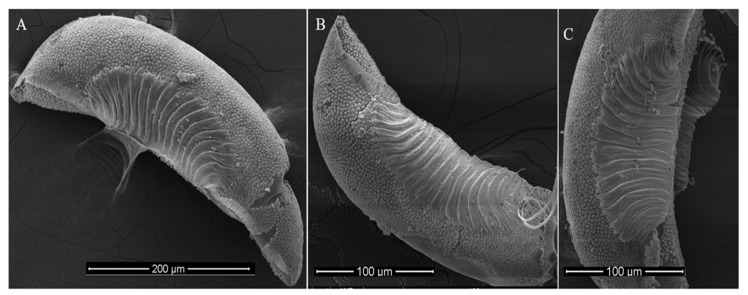
Scanning electron micrographs showing the egg ridge number in *Anopheles stephensi* “mysorensis”. (**A**) Lateral aspect (scale bar: 200 μm). (**B**) Lateral aspect (scale bar: 100 μm). (**C**) Ventral aspect showing floats and ribs (scale bar: 50 μm).

Approximately 900 eggs (300 per district) were analyzed for ridge count using stereomicroscopy at 40× magnification. Eggs were collected from multiple ovitraps placed at various locations and sampled at different times throughout the sampling period across each district, ensuring they represented a diverse population of *An. stephensi* females. This approach minimized the risk of overrepresentation from a small number of females. A uniform ridge count of 13–15 per egg was observed (Figure 2), identifying the mysorensis form of *An. stephensi* in the region.

### 3.2. Molecular Analysis

#### 3.2.1. Cytochrome Oxidase Subunit I (COI)

**Figure 3:**
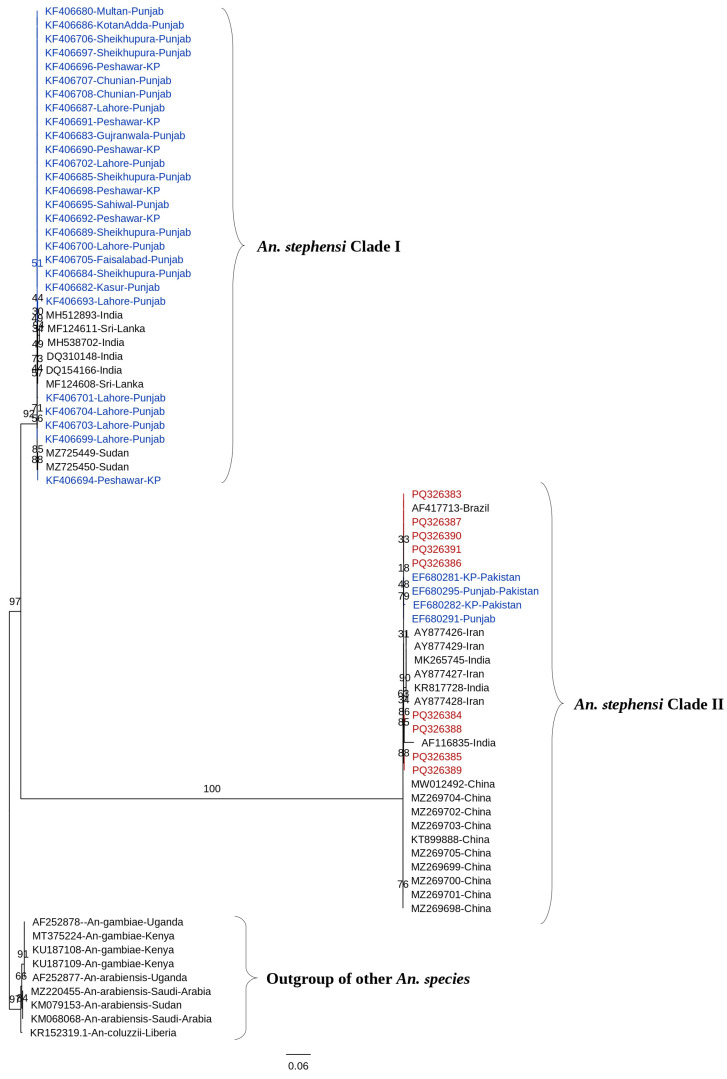
A maximum likelihood (ML) phylogenetic tree was constructed using 67 *An. stephensi* COI sequences from Pakistan and other global regions. The tree, rooted with outgroups of other *Anopheles* species, confirmed *An. stephensi* monophyly and identified two internal clades. Bootstrap support values, based on 1000 replicates, are displayed at each node, indicating the percentage of replicate trees in which the associated taxa clustered together. Branches were further evaluated using Shimodaira–Hasegawa-like approximate likelihood ratio tests (SH-like aLRT) with 1000 replicates. Evolutionary distances were calculated using the best-fit substitution model TN + F. The phylogenetic tree was visualized and refined using FigTree v1.4. Previously published COI sequences are marked in blue, while sequences generated in the current study are highlighted in red.

The current study sequenced a total of 17 isolates selected based on their geographic distribution across diverse ecological regions of KP to ensure representative genetic diversity (details provided in Table 1). The lengths of the COI, COII, and ITS2 fragments were 877 bp, 650 bp, and 650 bp, respectively. The COI sequences were trimmed to 758 bp for sequence similarity analysis using nucleotide BLAST (NCBI) and subsequent phylogenetic analysis. The *An. stephensi* COI sequences generated in this study from Pakistan exhibited a sequence similarity range of 99.74% to 100%. Out of 100 *An. stephensi* COI sequences available in GenBank, 58 sequences originating from Brazil, China, India, Iran, Sudan, and Sri Lanka matched the sequences generated in this study and were included in the analysis. Sequence similarity with Iranian and Indian sequences was 99.08%, while similarity with the Chinese lab strain and Brazilian *An. stephensi* COI sequences was 99.74% and 99.87%, respectively. The sequences from this study showed 99.56% to 99.85% similarity with previously reported Pakistani *An. stephensi* sequences (EF680281, EF680282, EF680291, and EF680295). The ML tree for COI (Figure 3), rooted with outgroups *An. gambiae* and *An. funestus*, confirmed *An. stephensi* monophyly (bootstrap 100%). It revealed two distinct clades: Clade I (Punjab/KP, Sudan, Sri Lanka, India; bootstrap 92%) and Clade II (China, Iran, Brazil, and some Pakistani sequences; bootstrap 88%). Within Clade I, Punjab and KP sequences formed tight subclades (bootstrap 85%), reflecting regional genetic homogeneity, with sequences from Sudan and Sri Lanka clustering closely with Indian sequences. Clade II showed greater internal divergence, particularly among Chinese sequences, with Iranian sequences showing intermediate branches. The high sequence similarity (>99%) across all isolates supports their classification as *An. stephensi*, though the separation into two clades suggests potential population structure rather than species-level divergence, as reciprocal monophyly was not fully demonstrated.

#### 3.2.2. Cytochrome Oxidase Subunit II (COII)

**Figure 4:**
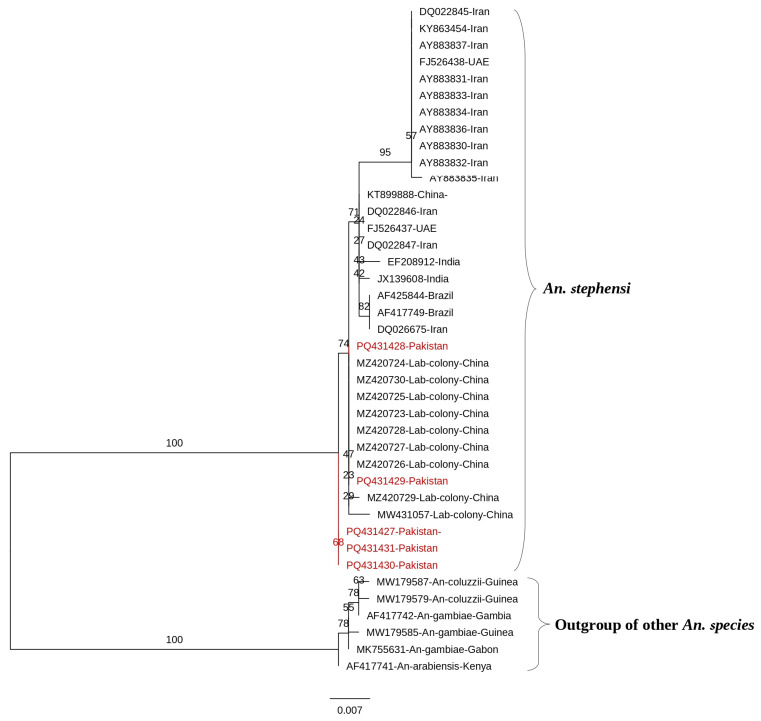
A maximum likelihood (ML) phylogenetic tree was constructed using 34 COII sequences of *An. stephensi* from Pakistan and other countries. The tree, rooted with outgroups of other *Anopheles* species, confirmed *An. stephensi* monophyly but revealed paraphyly with no clear geographic partitioning. Bootstrap support values, based on 1000 replicates, are displayed at each node, indicating the percentage of replicate trees in which the associated taxa clustered together. Branches were further evaluated using Shimodaira–Hasegawa-like approximate likelihood ratio tests (SH-like aLRT) with 1000 replicates. Evolutionary distances were calculated using the best-fit substitution model K3Pu + F+G4. The phylogenetic tree was visualized and refined using FigTree v1.4. The sequences generated in the current study are highlighted in red.

Our BLAST analysis identified 29 globally submitted COII sequences matching those generated in this study. The multiple sequence alignment of COII (560 bp) sequences from our study revealed a similarity range of 99.83% to 100% (Figure 4). The sequences from different countries with nucleotide variations have been shown in Supplementary File S1. The sequences from this study showed 99.46% to 100% similarity with Chinese sequences and 98.75% to 100% similarity with Iranian sequences. Those from the United Arab Emirates (UAE) and India exhibited similarity ranges of 99.28% to 99.64%, while sequences from Brazil and the USA demonstrated 99.46% similarity.

The phylogenetic analysis of *An. stephensi* COII sequences confirmed monophyly (bootstrap 95%) but showed no clear geographic partitioning. Pakistani sequences (highlighted in red) grouped into two clades, both closely related to a Chinese laboratory colony (bootstraps 74% and 68%). Brazilian sequences clustered with an Iranian sequence (bootstrap 82%), while other Iranian sequences were paraphyletic, appearing across multiple branches alongside Indian and UAE sequences. This paraphyly indicates a mixture of lineages, suggesting strong genetic connectivity within *An. stephensi* rather than distinct geographic structuring. The tree’s topology, with low to moderate bootstrap support for most nodes, supports the interpretation of a single species with high gene flow.

#### 3.2.3. Internal Transcribed Spacer 2

**Figure 5:**
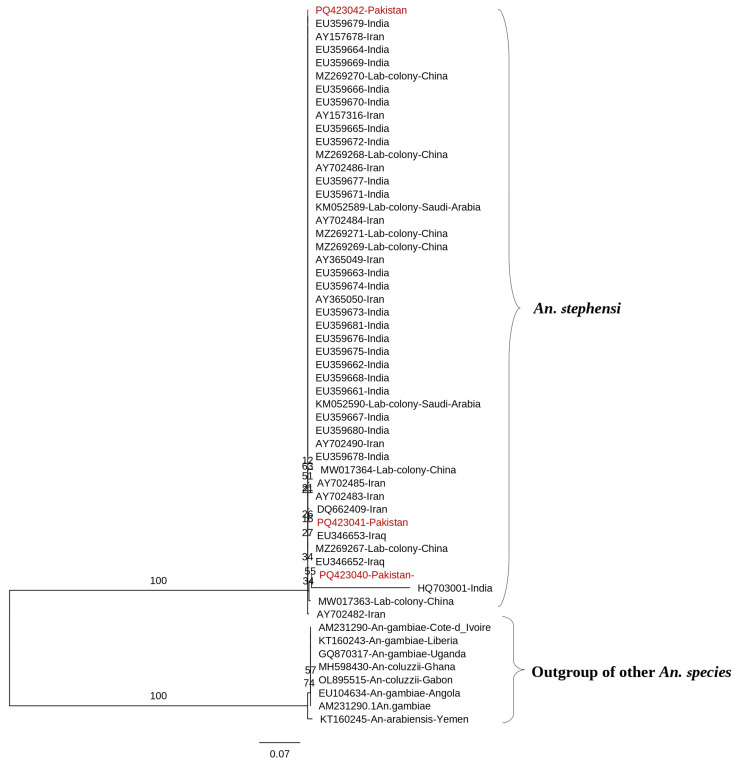
A maximum likelihood (ML) phylogenetic tree was constructed using 47 ITS2 sequences of *An. stephensi* from Pakistan and other countries. The tree, rooted with outgroups of other *Anopheles* species, confirmed *An. stephensi* monophyly but revealed a large polytomy with minimal regional structure. Bootstrap support values, based on 1000 replicates, are displayed at each node, indicating the percentage of replicate trees in which the associated taxa clustered together. Branches were further evaluated using Shimodaira–Hasegawa-like approximate likelihood ratio tests (SH-like aLRT) with 1000 replicates. Evolutionary distances were calculated using the best-fit substitution model, K2P. The phylogenetic tree was visualized and refined using FigTree v1.4. The sequences generated in the current study are highlighted in red.

BLAST analysis identified 44 globally submitted ITS2 sequences matching those generated in this study. Genetic similarity among the sequences ranged from 99.14% to 99.78%. The sequences showed 99.35% to 100% similarity with Iranian sequences. Comparison with Indian sequences from GenBank revealed a match of 99.35% to 100%. Alignment with Iraqi sequences (EU346652 and EU346653) ranged from 98% to 99%, with Saudi Arabian lab colony sequences from 99.35% to 100%, and with Chinese lab colony sequences from 98.42% to 100%. The phylogenetic analysis of all *An. stephensi* ITS2 sequences confirmed monophyly (bootstrap 98%) but revealed a large polytomy with minimal regional structure (Figure 5). Pakistani sequences (highlighted in red) formed two weakly supported clades: one allied with Indian and Chinese lab colony sequences (bootstrap 54%), and another with Iranian and Iraqi sequences (bootstrap 34%). Indian sequences exhibited broad diversity across multiple branches, while Iranian sequences included a highly divergent cluster (bootstrap 100%) and were otherwise intermingled with Indian, Saudi Arabian, and Chinese sequences (e.g., bootstrap 54% for a mixed clade with Indian, Saudi Arabian, and Chinese sequences; bootstrap 27% for another mixed clade). Iraqi sequences clustered alongside Iranian sequences in a weakly supported clade, contributing to the polytomy. Pakistani sequences, while forming weakly supported clades, were intermingled with sequences from other regions, reflecting a lack of distinct geographic clustering and suggesting extensive gene flow or incomplete lineage sorting.

### 3.3. Phylogeographic Dynamics of An. stephensi

#### 3.3.1. Cytochrome Oxidase I

**Figure 6:**
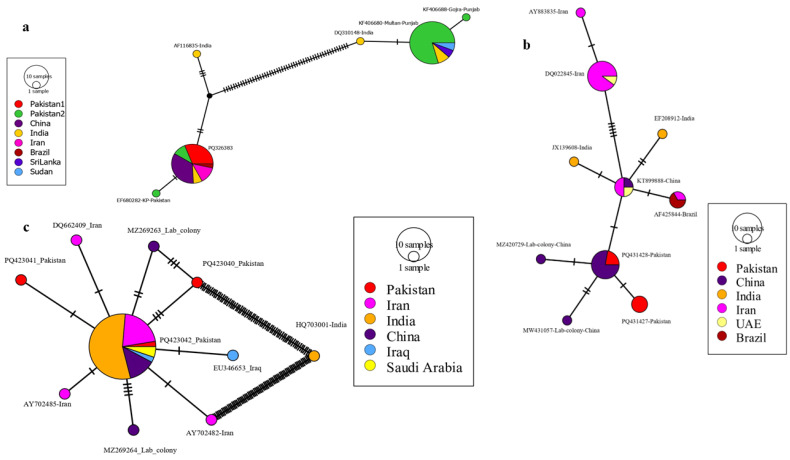
Phylogeographic network analysis of *An. stephensi* sequences was performed using the Minimum Spanning Network (MSN) approach in PopArt v1.7. The analysis included networks based on (**a**) COI, (**b**) COII, and (**c**) ITS2 sequences, incorporating globally available data. In these networks, nodes represent distinct haplotypes, with size proportional to sample number. Colors indicate geographic regions, and black dashes along connecting lines denote nucleotide substitutions between haplotypes. In the figure, “Pakistan 2” refers to sequences previously submitted from Punjab and KP provinces, while “Pakistan 1” denotes sequences generated in this study.

The phylogeographic network based on *An. stephensi* COI gene sequences (Figure 6a) revealed substantial haplotype diversity across geographic regions. Distinct haplotypes were represented as nodes, with node sizes reflecting the number of samples sharing each haplotype. Haplotypes from Pakistan showed greater connectivity, occupying a central position in the network. Specific haplotypes were shared between neighboring regions, such as India and Pakistan, indicating potential gene flow. The black dashes along the connecting lines represented nucleotide substitutions, with longer connections between certain regions (e.g., India and Sudan) indicating greater genetic divergence. This network highlights both regional clustering and inter-regional haplotype sharing within the COI gene.

#### 3.3.2. Cytochrome Oxidase II

The COII gene phylogeographic network (Figure 6b) revealed substantial haplotype diversity with clear geographic clustering. Distinct groups were formed by haplotypes from Pakistan and China, with laboratory colony samples from China clustering tightly. Connections among haplotypes from India, Brazil, and Iran suggested shared genetic ancestry or recent gene flow. The UAE sample clustered closely with Brazil, highlighting a possible genetic relationship between these geographically distant regions. The largest haplotypes were observed in Pakistan, with black dashes indicating moderate nucleotide substitutions between clusters.

#### 3.3.3. Internal Transcribed Spacer 2

The ITS2 phylogeographic network (Figure 6c) revealed high haplotype diversity, with the central haplotype predominantly represented by samples from Iran, indicating its ancestral or central role. Pakistani samples are represented by three haplotypes (PQ423040, PQ423041, PQ423042), with PQ423041 closely connected to the central haplotype, while PQ423040 and PQ423042 exhibit greater genetic divergence. The Indian haplotype (HQ703001) is highly divergent, separated from the central haplotype by a long branch with multiple mutational steps. The laboratory colony samples from China (MW017363 and MW017364) exhibit minimal divergence, clustering close to the central haplotype. The Saudi Arabian haplotype is directly connected to the central haplotype, while the Iraqi haplotype (EU346653) is one mutational step away. This network highlights significant genetic diversity and regional differentiation within *An. stephensi*.

### 3.4. Haplotypes Distribution

#### 3.4.1. COI-Based Network

The analysis of *An. stephensi* COI sequences identified six haplotypes distributed across multiple regions (Table 2). The most prevalent haplotype, Hap_2, represented 50.7% of the sequences and was widely distributed in Pakistan (27 sequences), Sudan (2), Sri Lanka (2), and China (3). Hap_1 was the second most frequent haplotype, accounting for 43.3% of the sequences, and was observed in Pakistan (12), Iran (4), India (2), China (10), and Brazil (1). Hap_3, Hap_4, Hap_5, and Hap_6 were less common, each contributing 1.5% to the total dataset. These minor haplotypes were confined to specific regions, including India (Hap_5 and Hap_6) and Pakistan (Hap_3 and Hap_4).

#### 3.4.2. COII-Based Network

The COII sequence analysis identified ten haplotypes, with Hap_7 being the most prevalent, accounting for 29.4% of the dataset (Table 2). This haplotype was observed in Pakistan (nine sequences), India (one), and the UAE (one). Hap_2 constituted 26.5% of the dataset and was found in Pakistan (two sequences) and China (seven sequences). Other haplotypes, including Hap_5 (11.8%) and Hap_6 (8.8%), were distributed across Iran, China, and Brazil. Haplotypes Hap_1, Hap_3, Hap_4, Hap_8, Hap_9, and Hap_10 were less common, each contributing less than 10% and primarily confined to specific regions.

#### 3.4.3. ITS2-Based Network

The ITS2 analysis identified 10 haplotypes, with Hap_3 being the most dominant, accounting for 80.8% of the sequences (Table 2). This haplotype was widely distributed across India (21 sequences), China (five), Iran (eight), and Saudi Arabia (two). Haplotypes Hap_1 and Hap_2 were exclusive to Pakistan, while Hap_4 to Hap_10 were sparsely distributed across Iran, Iraq, and China, each contributing 2.1% to the dataset.

### 3.5. An. stephensi Population Genetic Diversity

The population genetics analysis of *An. stephensi* COI, COII, and ITS2 nucleotide sequences revealed distinct patterns of genetic diversity and evolutionary dynamics across regions (Table 3). COI exhibited higher genetic variation, with Pakistan and India showing the greatest haplotype and nucleotide diversity. COII showed moderate diversity, with Iran, India, and Pakistan contributing to haplotype variation, while ITS2 displayed high haplotype diversity in Pakistan but comparatively lower nucleotide diversity across most regions.

Neutrality tests indicated contrasting evolutionary pressures (Table 3). Positive Tajima’s D and Fu’s Fs values for COI in Pakistan and India suggested balancing selection or population expansion. For COII, negative values in Iran and India indicated purifying selection or population contraction. Similarly, ITS2 showed negative Tajima’s D values for most regions, reflecting potential population contraction, with some regions, such as India, displaying positive Fu’s Fs values, suggesting population growth.

## 4. Discussion

This study provides valuable insights into the genetic diversity, population structure, and phylogeographic patterns of *An. stephensi*, a key malaria vector in South Asia. Our findings have significant implications for public health and vector control strategies, particularly in Pakistan, where *An. stephensi* is increasingly recognized as a major contributor to urban malaria transmission.

**Genetic Diversity and Population Structure**

Phylogenetic analysis of COI, COII, and ITS2 markers highlights the complex genetic landscape of *An. stephensi*, with implications for its adaptability and spread. The structured pattern observed in COI suggests potential population subdivision, which may reflect historical dispersal events or regional gene flow, particularly between Pakistan and India, as supported by their high genetic similarity [36,37,38,39,40,41]. Haplotype networks mirror this pattern, with COI showing structured haplotype diversity, while COII and ITS2 indicate greater connectivity across regions, consistent with the phylogenetic findings of population structure in COI and extensive gene flow in COII and ITS2. This connectivity aligns with *An. stephensi*’s known adaptability to diverse environments, as reported in studies from Afghanistan, Iran, and India, where the species thrives in both peri-urban and rural settings [7,8,12,26]. In contrast, the lack of geographic structure in COII and ITS2 points to extensive gene flow across regions, a pattern consistent with the species’ high dispersal capacity and human-mediated movement [37,40,41]. This genetic connectivity complicates the hypothesis of local adaptation or multiple introduction events, suggesting instead a more unified population structure driven by gene flow.

The predominance of the mysorensis form in KP is consistent with previous reports [19,20] and raises questions about its vectorial capacity in urbanizing regions, given its association with rural settings. Regional discrepancies in egg ridge counts [12,13,14,31,36] may reflect underlying genetic or environmental influences, warranting further investigation to understand their impact on vector competence. The marker-specific differences observed underscore COI’s utility for detecting population structure in anophelines, due to its rapid mutation rate and uniparental inheritance, which minimize recombination and allow for clearer lineage tracing [42,43]. Compared to other intraspecific analyses, such as microsatellites or whole-genome sequencing, COI offers a cost-effective and standardized approach for barcoding and population studies in anophelines, as demonstrated in other studies [42], which identified cryptic diversity in *An. stephensi*. However, the homogenized patterns in COII and ITS2 suggest that nuclear markers may be more influenced by gene flow, limiting their resolution for fine-scale structure in a dispersive species like *An. stephensi* [41,44]. The discordance across markers, potentially driven by mitochondrial introgression or nuclear recombination, highlights the need for genomic approaches to disentangle the roles of gene flow, incomplete lineage sorting, and selection in shaping *An. stephensi* populations.

**Phylogeographic Insights and Implications for Vector Control**

Haplotype network analysis highlights *An. stephensi*’s global expansion potential, with Pakistani populations centrally positioned among haplotypes from Sudan, Sri Lanka, and India, reflecting Pakistan’s biogeographic location at the Indo-Malayan and Palearctic confluence. This positioning, driven by extensive gene flow as seen in COII and ITS2, facilitates dispersal across regions, necessitating cross-border collaboration to address widespread transmission risks. The species’ adaptability to artificial water sources in urban settings [45,46,47,48] poses a significant challenge for malaria control, particularly in rapidly urbanizing regions [16,42,45]. This adaptability, coupled with genetic diversity, may accelerate the evolution of insecticide resistance or alter vector competence, heightening the risk of malaria transmission [49,50].

Given the predominance of the mysorensis form in KP, vector control strategies should be tailored accordingly. While this form primarily inhabits rural and peri-urban environments, its adaptation to urban settings necessitates integrated management approaches [41]. Targeted larval source reduction, focusing on irrigation channels, wells, and rice paddies, should be prioritized. Additionally, resistance monitoring and novel vector control strategies, including the Sterile Insect Technique (SIT) and Wolbachia-based biocontrol, could provide sustainable solutions to mitigate malaria transmission risks. Strengthening cross-border collaboration and genomic surveillance will be critical for monitoring *An. stephensi*’s spread and mitigating its impact on malaria transmission.

**Study Limitations and Future Directions**

Despite the valuable insights provided by this, certain limitations must be acknowledged. The relatively small sample size (17 isolates) constrains the generalizability of our findings, emphasizing the need for broader geographic sampling across malaria-endemic regions. Furthermore, the reliance on three molecular markers limits the resolution of genetic variation assessed. Future research should incorporate high-resolution genomic approaches, such as short tandem repeat (STR) markers and whole-genome sequencing, to refine our understanding of *An. stephensi* population structure, gene flow dynamics, and evolutionary trajectories [46].

From a public health perspective, these findings underscore the importance of region-specific vector control strategies tailored to *An. stephensi*’s ecological adaptability. While ITNs and IRS remain central to malaria control in rural and peri-urban settings [50,51], expanding genomic surveillance and ecological niche modeling will be critical for preemptively identifying emerging vector populations [41,51]. Strengthening cross-border collaboration and integrating advanced molecular surveillance techniques will be essential for mitigating *An. stephensi*’s role in malaria transmission and ensuring the long-term efficacy of control interventions.

## 5. Conclusions

This study highlights the growing challenges posed by *An. stephensi*’s expanding geographic range and genetic diversity, revealing marker-specific insights into its population dynamics. COI’s structured diversity suggests potential population subdivision, offering a valuable marker for tracking the species’ spread and targeting dominant haplotypes in control strategies. Conversely, the genetic connectivity indicated by COII and ITS2 emphasizes the need for regional and international collaboration in vector surveillance to address widespread gene flow. As *An. stephensi* continues to establish itself in urban and peri-urban environments, proactive and adaptive measures, including enhanced genomic surveillance, innovative control strategies, and coordinated global interventions, will be critical for mitigating its impact and addressing the growing threat of urban malaria.

## Figures and Tables

**Table 1:** Details of *An. stephensi* isolates collected from various locations, with ecological descriptions of each sampling site.

Sampling Site (Isolate)	District	Geographic Coordinates	Trap Location	Climate Zone	Annual Mean Temperature ( °C)	Annual Precipitation (mm)
P1	Peshawar	33.9437° N, 71.6199° E	Location 1			
P2		33.9982° N, 71.4862° E	Location 2			
P3		33.9744° N, 71.4359° E	Location 3			
P4		34.0156° N, 71.7127° E	Location 4	semi-arid	22.7	1110
P5		34.0259° N, 71.5601° E	Location 5			
P6		34.0054° N, 71.7237° E	Location 6			
M7	Mardan	34.3100° N, 72.0468° E	Location 1			
M8		34.1500° N, 72.0379° E	Location 2			
M9		34.2876° N, 71.9342° E	Location 3	hot semi-arid	22.2	1000
M10		34.3521° N, 72.0764° E	Location 4			
M11		34.3410° N, 72.2897° E	Location 5			
C12	Charsadda	34.1986° N, 71.7385° E	Location 1			
C13		34.3040° N, 71.6555° E	Location 2			
C14		34.2186° N, 71.5546° E	Location 3	semi-arid	19.74	1000
C15		34°16′47 N, 71°33′59° E	Location 4			
C16		34.1435° N, 71.7370° E	Location 5			
C17		34°19′7° N, 71°35′35° E	Location 6			

**Table 2:** Distribution of *An. stephensi* haplotypes across different countries in our analysis.

COI-based Haplotypes Distribution									
Haplotypes	Pakistan	Iran	India	China	Brazil	Sudan	Sri Lanka	Total	% Prevalence
Hap_1	12	4	2	10	1	0	0	29	43.3
Hap_2	27	0	3	0	0	2	2	34	50.7
Hap_3	1	0	0	0	0	0	0	1	1.5
Hap_4	1	0	0	0	0	0	0	1	1.5
Hap_5	0	0	1	0	0	0	0	1	1.5
Hap_6	0	0	1	0	0	0	0	1	1.5
**COII-based Haplotypes Distribution**									
**Haplotypes**	**Pakistan**	**Iran**	**India**	**China**	**Brazil**	**UAE**	**-**	**Total**	**% Prevalence**
Hap_1	3	0	0	0	0	0	-	3	8.8
Hap_2	2	0	0	7	0	0	-	9	26.5
Hap_3	0	0	0	1	0	0	-	1	2.9
Hap_4	0	0	0	1	0	0	-	1	2.9
Hap_5	0	2	0	1	0	1	-	4	11.8
Hap_6	0	1	0	0	2	0	-	3	8.8
Hap_7	0	9	0	0	0	1	-	10	29.4
Hap_8	0	1	0	0	0	0	-	1	2.9
Hap_9	0	0	1	0	0	0	-	1	2.9
Hap_10	0	0	1	0	0	0	-	1	2.9
**ITS2-based Haplotypes Distribution**									
**Haplotypes**	**Pakistan**	**Iran**	**India**	**China**	**Iraq**	**Saudi Arabia**		**Total**	**% Prevalence**
Hap_1	1	0	0	0	0	0	-	1	2.1
Hap_2	1	0	0	0	0	0	-	1	2.1
Hap_3	1	8	21	5	1	2	-	38	80.8
Hap_4	0	0	0	1	0	0	-	1	2.1
Hap_5	0	0	0	1	0	0	-	1	2.1
Hap_6	0	1	0	0	0	0	-	1	2.1
Hap_7	0	1	0	0	0	0	-	1	2.1
Hap_8	0	0	0	0	1	0	-	1	2.1
Hap_9	0	1	0	0	0	0	-	1	2.1
Hap_10	0	0	1	0	0	0	-	1	2.1

**Table 3:** Summary of genetic diversity indices and neutrality tests (Fu’s Fs and Tajima’s D) in *An. stephensi* populations based on COI, COII, and ITS2 sequences.

Cytochrome Oxidase I							
Variable	Pakistan	Iran	India	China	Brazil	Sudan	Sri Lanka
Number of sequences (*n*)	41	4	7	10	1	2	2
Number of segregating sites	43	0	47	0	0	0	0
Number of haplotypes (h)	4	1	4	1	1	1	1
Haplotype diversity (Hd)	0.49146	0	0.80	0	0	0	0
Nucleotide diversity (Pi)	0.16555	0	0.22587	0	0	0	0
k	18.70732	0	25.5238	0	n.d. *	0	0
Tajima’s D	3.05205	n.d.	2.00315	n.d.	n.d.	n.d.	n.d.
Fu’s Fs	43.549	n.d.	10.026	n.d.	n.d.	n.d.	n.d.
**Cytochrome Oxidase II**							
**Variable**	**Pakistan**	**Iran**	**India**	**China**	**Brazil**	**UAE**	**-**
Number of sequences (n)	5	13	2	10	2	2	-
Number of segregating sites	1	7	3	4	0	5	-
Number of haplotypes (h)	2	4	2	4	1	2	-
Haplotype diversity (Hd)	0.6	0.526	1	0.533	0	1	-
Nucleotide diversity (Pi)	0.00107	0.00398	0.00536	0.00143	0	0.00893	-
k	0.6	2.231	3	0.8	0	5	-
Tajima’s D	1.22474	−0.042	n.d.	−1.6671	n.d.	n.d.	-
Fu’s Fs	0.626	1.343	n.d.	−1.345	n.d.	n.d.	-
**Internal Transcribed Spacer 2**							
**Variable**	**Pakistan**	**Iran**	**India**	**China**	**Iraq**	**Saudi Arabia**	**-**
Number of sequences (n)	3	11	22	7	2	2	-
Number of segregating sites	4	3	71	6	1	0	-
Number of haplotypes (h)	3	4	2	3	2	1	-
Haplotype diversity (Hd)	1	0.491	0.091	0.524	1	0	-
Nucleotide diversity (Pi)	0.00572	0.00117	0.01385	0.00368	0.00215	0	-
k	2.667	0.545	6.455	1.714	1	0	-
Tajima’s D	n.d.	−1.6	−2.6719	−1.5241	n.d.	n.d.	-
Fu’s Fs	n.d.	−2.042	13.242	1.014	n.d.	n.d.	-

* n.d.: not determined.

## Data Availability

The study’s original findings are documented in the article/Supplementary Material, and any additional inquiries can be directed to the corresponding authors.

## Supplementary Materials

The following supporting information can be downloaded at https://www.mdpi.com/article/10.3390/tropicalmed10040109/s1. File S1: Table S1: List of sequences utilized in our analysis, comprising both NCBI-acquired sequences and those generated during this study. File S2: Multiple sequence alignment of the COI gene, highlighting nucleotide sequence variations across analyzed samples. File S3: Multiple sequence alignment of the COII gene, detailing nucleotide sequence variations across analyzed samples. File S4: Multiple sequence alignment of the ITS2 gene, illustrating nucleotide sequence variations across analyzed samples.

## References

[1] WHO World Malaria Report 2024. https://www.who.int/teams/global-malaria-programme/reports/world-malaria-report-2024.

[2] WHO (2024). Surveillance and Control of Anopheles stephensi: Country Experiences.

[3] Ministry of Health Pakistan (2022). Epidemiology of Malaria in Pakistan. https://www.who.int/emergencies/disease-outbreak-news/item/2022-DON413.

[4] Taylor R., Messenger L.A., Abeku T.A., Clarke S.E., Yadav R.S., Lines J. (2024). Invasive *Anopheles stephensi* in Africa: Insights from Asia. Trends Parasitol..

[5] Thomas S., Ravishankaran S., Justin N.A., Asokan A., Mathai M.T., Valecha N., Montgomery J., Thomas M.B., Eapen A. (2017). Resting and feeding preferences of *Anopheles stephensi* in an urban setting, perennial for malaria. Malar. J..

[6] Djadid N.D., Gholizadeh S., Aghajari M., Zehi A.H., Raeisi A., Zakeri S. (2006). Genetic analysis of rDNA-ITS2 and RAPD loci in field populations of the malaria vector, *Anopheles stephensi* (Diptera: Culicidae): Implications for the control program in Iran. Acta Trop..

[7] Oshaghi M., Yaghoobi F., Vatandoost H., Abai M., Akbarzadeh K. (2006). *Anopheles stephensi* biological forms, geographical distribution, and malaria transmission in malarious regions in Iran. Pak. J. Biol. Sci..

[8] Firooziyan S., Djadid N.D., Gholizadeh S. (2018). Speculation on the possibility for introducing *Anopheles stephensi* as a species complex: Preliminary evidence based on odorant-binding protein 1 intron I sequence. Malar. J..

[9] Gholizadeh S., Firooziyan S., Ladonni H., Hajipirloo H.M., Djadid N.D., Hosseini A., Raz A. (2015). The *Anopheles stephensi* odorant binding protein 1 (AsteObp1) gene: A new molecular marker for biological forms diagnosis. Acta Trop..

[10] Gholizadeh S., Zakeri S., Djadid N.D. (2013). Genotyping Plasmodium vivax isolates infecting *Anopheles stephensi*, an Asian main malaria vector. Exp. Parasitol..

[11] Gholizadeh S., Djadid N.D., Nouroozi B., Bekmohammadi M. (2013). Molecular phylogenetic analysis of Anopheles and Cellia subgenus anophelines (Diptera: Culicidae) in temperate and tropical regions of Iran. Acta Trop..

[12] Subbarao S.K., Vasantha K., Adak T., Sharma V.P., Curtis C.F. (1987). Egg float ridge number in *Anopheles stephensi*: Ecological variation and genetic analysis. Med. Vet. Entomol..

[13] Sweet W.C., Rao B.A. (1937). Races of *Anopheles stephensi* Liston. Indian Med. Gaz..

[14] Rao B.A., Sweet W.C., Subbarao A.M. (1938). Ova measurements of *Anopheles stephensi* type and *Anopheles stephensi* var. mysorensis. J. Malar. Inst. India.

[15] Khan J., Gholizadeh S., Zhang D., Wang G., Guo Y., Zheng X., Wu Z., Wu Y. (2022). Identification of a biological form in the *Anopheles stephensi* laboratory colony using the odorant-binding protein 1 intron I sequence. PLoS ONE.

[16] Sinka M.E., Bangs M.J., Manguin S., Chareonviriyaphap T., Patil A.P., Temperley W.H., Gething P.W., Elyazar I.R., Kabaria C.W., Harbach R.E. (2011). The dominant Anopheles vectors of human malaria in the Asia-Pacific region: Occurrence data, distribution maps and bionomic precis. Parasit. Vectors.

[17] Neafsey D.E., Waterhouse R.M., Abai M.R., Aganezov S.S., Alekseyev M.A., Allen J.E., Amon J., Arcà B., Arensburger P., Artemov G. (2015). Highly evolvable malaria vectors: The genomes of 16 Anopheles mosquitoes. Science.

[18] Yared S., Gebressielasie A., Damodaran L., Bonnell V., Lopez K., Janies D., Carter T.E. (2020). Insecticide resistance in *Anopheles stephensi* in Somali Region, eastern Ethiopia. Malar. J..

[19] Khan M.A., Aaqil M., Hafeez M. (1972). Genetic and morphological variations in a national population of the malaria mosquito, *Anopheles stephensi* from Karachi, Pakistan. Biologia.

[20] Afridi M.K., Talibi S.A., Rashid S.A., Hussain M.Z.Y. (1958). Identification of races of *Anopheles stephensi* prevalent in the federal Karachi area by measurement of their ova. Pak. J. Health.

[21] Klinkenberg E., Konradsen F., Herrel N., Mukhtar M., van der Hoek W., Amerasinghe F.P. (2004). Malaria vectors in the changing environment of the southern Punjab, Pakistan. Trans. R. Soc. Trop. Med. Hyg..

[22] Khan J., Adil M., Wang G., Tsheten T., Zhang D., Pan W., Khan M.A., Rehman I.U., Zheng X., Wu Z. (2022). A cross-sectional study to assess the epidemiological situation and associated risk factors of dengue fever; knowledge, attitudes, and practices about dengue prevention in Khyber Pakhtunkhwa Province, Pakistan. Front. Public Health.

[23] Malik S.M., Awan H., Khan N. (2012). Mapping vulnerability to climate change and its repercussions on human health in Pakistan. Glob. Health.

[24] Pakistan Meteorological Department. https://rmckpk.pmd.gov.pk/.

[25] Ali N., Noreen S., Khan K., Wahid S. (2015). Population dynamics of mosquitoes and malaria vector incrimination in district Charsadda, Khyber Pakhtunkhwa (KP), Pakistan. Acta Trop..

[26] Rowland M., Hewitt S., Durrani N., Bano N., Wirtz R. (1997). Transmission and control of vivax malaria in Afghan refugee settlements in Pakistan. Trans. R. Soc. Trop. Med. Hyg..

[27] Zheng X., Zhang D., Li Y., Wu Y., Liang K., Liang X., Liang Y., Pan X., Hu L., Sun Q. (2019). Incompatible and sterile insect techniques combined eliminate mosquitoes. Nature.

[28] Amerasinghe F.P., Mukhtar M., Herrel N. (2002). Keys to the Anopheline mosquitoes (Diptera: Culicidae) of Pakistan. J. Med. Entomol..

[29] Altschul S.F., Gish W., Miller W., Myers E.W., Lipman D.J. (1990). Basic local alignment search tool. J. Mol. Biol..

[30] Larkin M.A., Blackshields G., Brown N.P., Chenna R., McGettigan P.A., McWilliam H., Valentin F., Wallace I.M., Wilm A., Lopez R. (2007). ClustalW and ClustalX version 2. Bioinformatics.

[31] Tamura K., Peterson D., Peterson N., Stecher G., Nei M., Kumar S. (2011). MEGA5: Molecular evolutionary genetics analysis using maximum likelihood, evolutionary distance, and maximum parsimony methods. Mol. Biol. Evol..

[32] Nguyen L.T., Schmidt H.A., von Haeseler A., Minh B.Q. (2015). IQ-TREE: A fast and effective stochastic algorithm for estimating maximum-likelihood phylogenies. Mol. Biol. Evol..

[33] Hoang D.T., Chernomor O., von Haeseler A., Minh B.Q., Vinh L.S. (2018). UFBoot2: Improving the ultrafast bootstrap approximation. Mol. Biol. Evol..

[34] Leigh J.W., Bryant D. (2015). PopArt: Full-feature software for haplotype network construction. Methods Ecol. Evol..

[35] Rozas J., Ferrer-Mata A., Sánchez-DelBarrio J.C., Guirao-Rico S., Librado P., Ramos-Onsins S.E., Sánchez-Gracia A. (2017). DnaSP 6: DNA sequence polymorphism analysis of large data sets. Mol. Biol. Evol..

[36] Nagpal B.N., Srivastava A., Kalra N.L., Subbarao S.K. (2003). Spiracular indices in *Anopheles stephensi*: A taxonomic tool to identify ecological variants. J. Med. Entomol..

[37] Abubakr M., Sami H., Mahdi I., Altahir O., Abdelbagi H., Mohamed N.S., Ahmed A. (2022). The phylodynamic and spread of the invasive Asian malaria vectors, *Anopheles stephensi*, in Sudan. Biology.

[38] Gakhar S.K., Sharma R., Sharma A. (2013). Population genetic structure of malaria vector *Anopheles stephensi* Liston (Diptera: Culicidae). Indian J. Exp. Biol..

[39] Alam M.Z., Niaz Arifin S.M., Al-Amin H.M., Alam M.S., Rahman M.S. (2017). A spatial agent-based model of *Anopheles vagus* for malaria epidemiology: Examining the impact of vector control interventions. Malar. J..

[40] Sharma R., Sharma A., Kumar A., Dube M., Gakhar S.K. (2016). Population genetic structure of urban malaria vector *Anopheles stephensi* in India. Infect. Genet. Evol..

[41] Sinka M.E., Pironon S., Massey N.C., Longbottom J., Hemingway J., Moyes C.L., Willis K.J. (2020). A new malaria vector in Africa: Predicting the expansion range of and identifying the urban populations at risk. Proc. Natl. Acad. Sci. USA.

[42] Carter T.E., Yared S., Getachew D., Spear J., Choi S.H., Samake J.N., Mumba P., Dengela D., Yohannes G., Chibsa S. (2021). Genetic diversity of *Anopheles stephensi* in Ethiopia provides insight into patterns of spread. Parasit. Vectors.

[43] Beebe N.W. (2018). DNA barcoding mosquitoes: Advice for potential prospectors. Parasitology.

[44] Hillis D.M., Dixon M.T. (1991). Ribosomal DNA: Molecular evolution and phylogenetic inference. Q. Rev. Biol..

[45] Thakare A., Ghosh C., Alalamath T., Kumar N., Narang H., Whadgar S., Paul K., Shrotri S., Kumar S., Soumya M. (2022). The genome trilogy of *Anopheles stephensi*, an urban malaria vector, reveals structure of a locus associated with adaptation to environmental heterogeneity. Sci. Rep..

[46] Subbarao S.K., Nanda N., Rahi M., Raghavendra K. (2019). Biology and bionomics of malaria vectors in India: Existing information and what more needs to be known for strategizing elimination of malaria. Malar. J..

[47] Dharmasiri G.A., Perera A.Y., Harishchandra J., Herath H., Aravindan K., Jayasooriya H.T.R., Ranawaka G.R., Hewavitharane M. (2017). First record of Anopheles stephensi in Sri Lanka: A potential challenge for prevention of malaria reintroduction. Malar. J..

[48] Balkew M., Mumba P., Yohannes G., Abiy E., Getachew D., Yared S., Worku A., Gebresilassie A., Tadesse F.G., Gadisa E. (2021). An update on the distribution, bionomics, and insecticide susceptibility of *Anopheles stephensi* in Ethiopia, 2018–2020. Malar. J..

[49] Surendran S.N., Sivabalakrishnan K., Gajapathy K., Arthiyan S., Jayadas T.T., Karvannan K., Raveendran S., Parakrama Karunaratne S.H.P., Ramasamy R. (2018). Genotype and biotype of invasive *Anopheles stephensi* in Mannar Island of Sri Lanka. Parasit. Vectors.

[50] Teshome A., Erko B., Golassa L., Yohannes G., Irish S.R., Zohdy S., Yoshimizu M., Dugassa S. (2023). Resistance of *Anopheles stephensi* to selected insecticides used for indoor residual spraying and long-lasting insecticidal nets in Ethiopia. Malar. J..

[51] Mnzava A., Monroe A.C., Okumu F. (2022). *Anopheles stephensi* in Africa requires a more integrated response. Malar. J..

