# Supplementary material for: Phylogeographic Patterns and Genetic Diversity of Anopheles stephensi: Implications for Global Malaria Transmission
Source: Trop Med Infect Dis. 2025 Apr 16;10(4):109. doi: 10.3390/tropicalmed10040109 (PMC12031451; doi:10.3390/tropicalmed10040109)
Supplement: Supplementary file 1 [file tropicalmed-10-00109-s001.zip › Supplementary File S1.pdf]

Table S1: Summary of the sequences used in our analysis.

COI (758 bp)		COII (560bp)		ITS2 (472bp)	
**GenBank accessions numbers**	**The closest available public sequence (n=16)**	**GenBank accessions numbers**	**The closest available public sequence (n=29)**	**GenBank accessions numbers**	**The closest available public sequence (n=44)**
1. PQ326383<br>2. PQ326384<br>3. PQ326385<br>4. PQ326386<br>5. PQ326387<br>6. PQ326388<br>7. PQ326389<br>8. PQ326390<br>9. PQ326391	MZ269698-MZ269705<br>AY877426-AY877429<br>KT899888<br>MW012492<br>AF417713<br>KR817728	1. PQ431427<br>2. PQ431428<br>3. PQ431429<br>4. PQ431430<br>5. PQ431431	AY883830-AY883837<br>MZ420723-MZ420730<br>DQ022845-DQ022847<br>MW431057<br>KT899888<br>AF425844<br>AF417749<br>DQ026675<br>KY863454<br>EF208912<br>FJ526438<br>FJ526437<br>JX139608	1. PQ423040<br>2. PQ423041<br>3. PQ423042	MZ269267-MZ269271<br>EU359661-EU359681<br>AY702482-AY702486<br>KM052589<br>KM052590<br>AY365050<br>AY365049<br>AY702490<br>AY157316<br>AY157678<br>EU346653<br>EU346652<br>DQ662409<br>HQ703001<br>MW017364<br>MW017363
