# Supplementary material for: Phylogeographic Patterns and Genetic Diversity of Anopheles stephensi: Implications for Global Malaria Transmission
Source: Trop Med Infect Dis. 2025 Apr 16;10(4):109. doi: 10.3390/tropicalmed10040109 (PMC12031451; doi:10.3390/tropicalmed10040109)
Supplement: Supplementary file 1 [file tropicalmed-10-00109-s001.zip › Supplementary File S2 COI Sequences.pdf]

```
#PQ326383     TCA CAC ATT ATT ACT CAA GAA AGA GGT AAA AAG GAA ACA TTC GGA AAT TTA GGA ATA ATT  [ 60]
#PQ326384     ... ... ... ... ... ... ... ... ... ... ... ... ... ... ... ... ... ... ... ...  [ 60]
#PQ326385     ... ... ... ... ... ... ... ... ... ... ... ... ... ... ... ... ... ... ... ...  [ 60]
#PQ326386     ... ... ... ... ... ... ... ... ... ... ... ... ... ... ... ... ... ... ... ...  [ 60]
#PQ326387     ... ... ... ... ... ... ... ... ... ... ... ... ... ... ... ... ... ... ... ...  [ 60]
#PQ326388     ... ... ... ... ... ... ... ... ... ... ... ... ... ... ... ... ... ... ... ...  [ 60]
#PQ326389     ... ... ... ... ... ... ... ... ... ... ... ... ... ... ... ... ... ... ... ...  [ 60]
#PQ326390     ... ... ... ... ... ... ... ... ... ... ... ... ... ... ... ... ... ... ... ...  [ 60]
#PQ326391     ... ... ... ... ... ... ... ... ... ... ... ... ... ... ... ... ... ... ... ...  [ 60]
#MW012492     ... ... ... ... ... ... ... ... ... ... ... ... ... ... ... ... ... ... ... ...  [ 60]
#MZ269698     ... ... ... ... ... ... ... ... ... ... ... ... ... ... ... ... ... ... ... ...  [ 60]
#MZ269699     ... ... ... ... ... ... ... ... ... ... ... ... ... ... ... ... ... ... ... ...  [ 60]
#MZ269700     ... ... ... ... ... ... ... ... ... ... ... ... ... ... ... ... ... ... ... ...  [ 60]
#MZ269701     ... ... ... ... ... ... ... ... ... ... ... ... ... ... ... ... ... ... ... ...  [ 60]
#MZ269702     ... ... ... ... ... ... ... ... ... ... ... ... ... ... ... ... ... ... ... ...  [ 60]
#MZ269703     ... ... ... ... ... ... ... ... ... ... ... ... ... ... ... ... ... ... ... ...  [ 60]
#MZ269704     ... ... ... ... ... ... ... ... ... ... ... ... ... ... ... ... ... ... ... ...  [ 60]
#MZ269705     ... ... ... ... ... ... ... ... ... ... ... ... ... ... ... ... ... ... ... ...  [ 60]
#KT899888     ... ... ... ... ... ... ... ... ... ... ... ... ... ... ... ... ... ... ... ...  [ 60]
#AY877426     ... ... ... ... ... ... ... ... ... ... ... ... ... ... ... ... ... ... ... ...  [ 60]
#AY877428     ... ... ... ... ... ... ... ... ... ... ... ... ... ... ... ... ... ... ... ...  [ 60]
#AY877429     ... ... ... ... ... ... ... ... ... ... ... ... ... ... ... ... ... ... ... ...  [ 60]
#AY877427     ... ... ... ... ... ... ... ... ... ... ... ... ... ... ... ... ... ... ... ...  [ 60]
#AF417713     ... ... ... ... ... ... ... ... ... ... ... ... ... ... ... ... ... ... ... ...  [ 60]
#KR817728     ... ... ... ... ... ... ... ... ... ... ... ... ... ... ... ... ... ... ... ...  [ 60]

#PQ326383     TAT GCT ATA TTA GCA ATT GGA TTA CTT GGA TTT ATC GTA TGA GCC CAC CAT ATG TTT ACA  [120]
#PQ326384     ... ... ... ... ... ... ... ... ... ... ... ... ... ... ... ... ... ... ... ...  [120]
#PQ326385     ... ... ... ... ... ... ... ... ... ... ... ... ... ... ... ... ... ... ... ...  [120]
#PQ326386     ... ... ... ... ... ... ... ... ... ... ... ... ... ... ... ... ... ... ... ...  [120]
#PQ326387     ... ... ... ... ... ... ... ... ... ... ... ... ... ... ... ... ... ... ... ...  [120]
#PQ326388     ... ... ... ... ... ... ... ... ... ... ... ... ... ... ... ... ... ... ... ...  [120]
#PQ326389     ... ... ... ... ... ... ... ... ... ... ... ... ... ... ... ... ... ... ... ...  [120]
#PQ326390     ... ... ... ... ... ... ... ... ... ... ... ... ... ... ... ... ... ... ... ...  [120]
#PQ326391     ... ... ... ... ... ... ... ... ... ... ... ... ... ... ... ... ... ... ... ...  [120]
#MW012492     ... ... ... ... ... ... ... ... ... ... ... ... ... ... ... ... ... ... ... ...  [120]
#MZ269698     ... ... ... ... ... ... ... ... ... ... ... ... ... ... ... ... ... ... ... ...  [120]
#MZ269699     ... ... ... ... ... ... ... ... ... ... ... ... ... ... ... ... ... ... ... ...  [120]
#MZ269700     ... ... ... ... ... ... ... ... ... ... ... ... ... ... ... ... ... ... ... ...  [120]
#MZ269701     ... ... ... ... ... ... ... ... ... ... ... ... ... ... ... ... ... ... ... ...  [120]
#MZ269702     ... ... ... ... ... ... ... ... ... ... ... ... ... ... ... ... ... ... ... ...  [120]
#MZ269703     ... ... ... ... ... ... ... ... ... ... ... ... ... ... ... ... ... ... ... ...  [120]
#MZ269704     ... ... ... ... ... ... ... ... ... ... ... ... ... ... ... ... ... ... ... ...  [120]
#MZ269705     ... ... ... ... ... ... ... ... ... ... ... ... ... ... ... ... ... ... ... ...  [120]
#KT899888     ... ... ... ... ... ... ... ... ... ... ... ... ... ... ... ... ... ... ... ...  [120]
#AY877426     ... ... ... ... ... ... ... ... ... ... ... ..T ... ... ... ... ... ... ... ...  [120]
#AY877428     ... ... ... ... ... ... ... ... ... ... ... ..T ... ... ... ... ... ... ... ...  [120]
#AY877429     ... ... ... ... ... ... ... ... ... ... ... ..T ... ... ... ... ... ... ... ...  [120]
#AY877427     ... ... ... ... ... ... ... ... ... ... ... ..T ... ... ... ... ... ... ... ...  [120]
#AF417713     ... ... ... ... ... ... ... ... ... ... ... ... ... ... ... ... ... ... ... ...  [120]
#KR817728     ... ... ... ... ... ... ... ... ... ... ... ..T ... ... ... ... ... ... ... ...  [120]

#PQ326383     GTA GGA ATA GAC GTA GAT ACT CGA GCT TAT TTT ACA TCA GCT ACA ATA ATT ATT GCT GTT  [180]
#PQ326384     ... ... ... ... ... ... ... ... ... ... ... ... ... ... ... ... ... ... ... ...  [180]
#PQ326385     ... ... ... ... ... ... ... ... ... ... ... ... ... ... ... ... ... ... ... ...  [180]
#PQ326386     ... ... ... ... ... ... ... ... ... ... ... ... ... ... ... ... ... ... ... ...  [180]
#PQ326387     ... ... ... ... ... ... ... ... ... ... ... ... ... ... ... ... ... ... ... ...  [180]
#PQ326388     ... ... ... ... ... ... ... ... ... ... ... ... ... ... ... ... ... ... ... ...  [180]
#PQ326389     ... ... ... ... ... ... ... ... ... ... ... ... ... ... ... ... ... ... ... ...  [180]
#PQ326390     ... ... ... ... ... ... ... ... ... ... ... ... ... ... ... ... ... ... ... ...  [180]
#PQ326391     ... ... ... ... ... ... ... ... ... ... ... ... ... ... ... ... ... ... ... ...  [180]
#MW012492     ... ... ... ... ... ... ... ... ... ... ... ... ... ... ... ... ... ... ... ...  [180]
#MZ269698     ... ... ... ... ... ... ... ... ... ... ... ... ... ... ... ... ... ... ... ...  [180]
#MZ269699     ... ... ... ... ... ... ... ... ... ... ... ... ... ... ... ... ... ... ... ...  [180]
#MZ269700     ... ... ... ... ... ... ... ... ... ... ... ... ... ... ... ... ... ... ... ...  [180]
#MZ269701     ... ... ... ... ... ... ... ... ... ... ... ... ... ... ... ... ... ... ... ...  [180]
```

```
#MZ269702    ... ... ... ... ... ... ... ... ... ... ... ... ... ... ... ... ... ... ... ...  [180]
#MZ269703    ... ... ... ... ... ... ... ... ... ... ... ... ... ... ... ... ... ... ... ...  [180]
#MZ269704    ... ... ... ... ... ... ... ... ... ... ... ... ... ... ... ... ... ... ... ...  [180]
#MZ269705    ... ... ... ... ... ... ... ... ... ... ... ... ... ... ... ... ... ... ... ...  [180]
#KT899888    ... ... ... ... ... ... ... ... ... ... ... ... ... ... ... ... ... ... ... ...  [180]
#AY877426    ... ... ... ... ... ... ... ... ... ... ... ... ... ... ... ... ... ... ... ...  [180]
#AY877428    ... ... ... ... ... ... ... ... ... ... ... ... ... ... ... ... ... ... ... ...  [180]
#AY877429    ... ... ... ... ... ... ... ... ... ... ... ... ... ... ... ... ... ... ... ...  [180]
#AY877427    ... ... ... ... ... ... ... ... ... ... ... ... ... ... ... ... ... ... ... ...  [180]
#AF417713    ... ... ... ... ... ... ... ... ... ... ... ... ... ... ... ... ... ... ... ...  [180]
#KR817728    ... ... ... ... ... ... ... ... ... ... ... ... ... ... ... ... ... ... ... ...  [180]

#PQ326383    CCA ACT GGA ATT AAA ATT TTT AGT TGA TTA GCT ACA TTA CAC GGA ACA CAA CTT ACT TAT  [240]
#PQ326384    ... ... ... ... ... ... ... ... ... ... ... ... ... ... ... ... ... ... ... ...  [240]
#PQ326385    ... ... ... ... ... ... ... ... ... ... ... ... ... ... ... ... ... ... ... ...  [240]
#PQ326386    ... ... ... ... ... ... ... ... ... ... ... ... ... ... ... ... ... ... ... ...  [240]
#PQ326387    ... ... ... ... ... ... ... ... ... ... ... ... ... ... ... ... ... ... ... ...  [240]
#PQ326388    ... ... ... ... ... ... ... ... ... ... ... ... ... ... ... ... ... ... ... ...  [240]
#PQ326389    ... ... ... ... ... ... ... ... ... ... ... ... ... ... ... ... ... ... ... ...  [240]
#PQ326390    ... ... ... ... ... ... ... ... ... ... ... ... ... ... ... ... ... ... ... ...  [240]
#PQ326391    ... ... ... ... ... ... ... ... ... ... ... ... ... ... ... ... ... ... ... ...  [240]
#MW012492    ... ... ... ... ... ... ... ... ... ... ..A ... ... ... ... ... ... ... ... ...  [240]
#MZ269698    ... ... ... ... ... ... ... ... ... ... ..A ... ... ... ... ... ... ... ... ...  [240]
#MZ269699    ... ... ... ... ... ... ... ... ... ... ..A ... ... ... ... ... ... ... ... ...  [240]
#MZ269700    ... ... ... ... ... ... ... ... ... ... ..A ... ... ... ... ... ... ... ... ...  [240]
#MZ269701    ... ... ... ... ... ... ... ... ... ... ..A ... ... ... ... ... ... ... ... ...  [240]
#MZ269702    ... ... ... ... ... ... ... ... ... ... ..A ... ... ... ... ... ... ... ... ...  [240]
#MZ269703    ... ... ... ... ... ... ... ... ... ... ..A ... ... ... ... ... ... ... ... ...  [240]
#MZ269704    ... ... ... ... ... ... ... ... ... ... ..A ... ... ... ... ... ... ... ... ...  [240]
#MZ269705    ... ... ... ... ... ... ... ... ... ... ..A ... ... ... ... ... ... ... ... ...  [240]
#KT899888    ... ... ... ... ... ... ... ... ... ... ..A ... ... ... ... ... ... ... ... ...  [240]
#AY877426    ... ... ... ... ... ... ... ... ... ... ... ... ... ... ... ... ... ... ... ...  [240]
#AY877428    ... ... ... ... ... ... ... ... ... ... ... ... ... ... ... ... ... ... ... ...  [240]
#AY877429    ... ... ... ... ... ... ... ... ... ... ... ... ... ... ... ... ... ... ... ...  [240]
#AY877427    ... ... ... ... ... ... ... ... ... ... ... ... ... ... ... ... ... ... ... ...  [240]
#AF417713    ... ... ... ... ... ... ... ... ... ... ... ... ... ... ... ... ... ... ... ...  [240]
#KR817728    ... ... ... ... ... ... ... ... ... ... ... ... ... ... ... ... ... ... ... ...  [240]

#PQ326383    AGC CCA GCT ATA TTA TGA GCA TTT GGA TTT GTA TTT TTA TTT ACA GTT GGA GGA TTA ACT  [300]
#PQ326384    ... ... ... ... ... ... ... ... ... ... ... ... ... ... ... ... ... ... ... ...  [300]
#PQ326385    ... ... ... ... ... ... ... ... ... ... ... ... ... ... ... ... ... ... ... ...  [300]
#PQ326386    ... ... ... ... ... ... ... ... ... ... ... ... ... ... ... ... ... ... ... ...  [300]
#PQ326387    ... ... ... ... ... ... ... ... ... ... ... ... ... ... ... ... ... ... ... ...  [300]
#PQ326388    ... ... ... ... ... ... ... ... ... ... ... ... ... ... ... ... ... ... ... ...  [300]
#PQ326389    ... ... ... ... ... ... ... ... ... ... ... ... ... ... ... ... ... ... ... ...  [300]
#PQ326390    ... ... ... ... ... ... ... ... ... ... ... ... ... ... ... ... ... ... ... ...  [300]
#PQ326391    ... ... ... ... ... ... ... ... ... ... ... ... ... ... ... ... ... ... ... ...  [300]
#MW012492    ... ... ... ... ... ... ... ... ... ... ... ... ... ... ... ... ... ... ... ...  [300]
#MZ269698    ... ... ... ... ... ... ... ... ... ... ... ... ... ... ... ... ... ... ... ...  [300]
#MZ269699    ... ... ... ... ... ... ... ... ... ... ... ... ... ... ... ... ... ... ... ...  [300]
#MZ269700    ... ... ... ... ... ... ... ... ... ... ... ... ... ... ... ... ... ... ... ...  [300]
#MZ269701    ... ... ... ... ... ... ... ... ... ... ... ... ... ... ... ... ... ... ... ...  [300]
#MZ269702    ... ... ... ... ... ... ... ... ... ... ... ... ... ... ... ... ... ... ... ...  [300]
#MZ269703    ... ... ... ... ... ... ... ... ... ... ... ... ... ... ... ... ... ... ... ...  [300]
#MZ269704    ... ... ... ... ... ... ... ... ... ... ... ... ... ... ... ... ... ... ... ...  [300]
#MZ269705    ... ... ... ... ... ... ... ... ... ... ... ... ... ... ... ... ... ... ... ...  [300]
#KT899888    ... ... ... ... ... ... ... ... ... ... ... ... ... ... ... ... ... ... ... ...  [300]
#AY877426    ... ... ... ... ... ... ... ... ... ... ... ... ... ... ... ... ... ... ... ...  [300]
#AY877428    ... ... ... ... ... ... ... ... ... ... ... ... ... ... ... ... ... ... ... ...  [300]
#AY877429    ... ... ... ... ... ... ... ... ... ... ... ... ... ... ... ... ... ... ... ...  [300]
#AY877427    ... ... ... ... ... ... ... ... ... ... ... ... ... ... ... ... ... ... ... ...  [300]
#AF417713    ... ... ... ... ... ... ... ... ... ... ... ... ... ... ... ... ... ... ... ...  [300]
#KR817728    ... ... ... ... ... ... ... ... ... ... ... ... ... ... ... ... ... ... ... ...  [300]

#PQ326383    GGA GTT GTT TTA GCA AAT TCA TCA ATT GAT ATT GTT TTA CAT GAT ACT TAT TAT GTT GTA  [360]
#PQ326384    ... ... ... ... ... ... ... ... ... ... ... ... ... ... ... ... ... ... ... ...  [360]
```

```
#PQ326385    ... ... ... ... ... ... ... ... ... ... ... ... ... ... ... ... ... ... ... ...  [360]
#PQ326386    ... ... ... ... ... ... ... ... ... ... ... ... ... ... ... ... ... ... ... ...  [360]
#PQ326387    ... ... ... ... ... ... ... ... ... ... ... ... ... ... ... ... ... ... ... ...  [360]
#PQ326388    ... ... ... ... ... ... ... ... ... ... ... ... ... ... ... ... ... ... ... ...  [360]
#PQ326389    ... ... ... ... ... ... ... ... ... ... ... ... ... ... ... ... ... ... ... ...  [360]
#PQ326390    ... ... ... ... ... ... ... ... ... ... ... ... ... ... ... ... ... ... ... ...  [360]
#PQ326391    ... ... ... ... ... ... ... ... ... ... ... ... ... ... ... ... ... ... ... ...  [360]
#MW012492    ... ... ... ... ... ... ... ... ... ... ... ... ... ... ... ... ... ... ... ...  [360]
#MZ269698    ... ... ... ... ... ... ... ... ... ... ... ... ... ... ... ... ... ... ... ...  [360]
#MZ269699    ... ... ... ... ... ... ... ... ... ... ... ... ... ... ... ... ... ... ... ...  [360]
#MZ269700    ... ... ... ... ... ... ... ... ... ... ... ... ... ... ... ... ... ... ... ...  [360]
#MZ269701    ... ... ... ... ... ... ... ... ... ... ... ... ... ... ... ... ... ... ... ...  [360]
#MZ269702    ... ... ... ... ... ... ... ... ... ... ... ... ... ... ... ... ... ... ... ...  [360]
#MZ269703    ... ... ... ... ... ... ... ... ... ... ... ... ... ... ... ... ... ... ... ...  [360]
#MZ269704    ... ... ... ... ... ... ... ... ... ... ... ... ... ... ... ... ... ... ... ...  [360]
#MZ269705    ... ... ... ... ... ... ... ... ... ... ... ... ... ... ... ... ... ... ... ...  [360]
#KT899888    ... ... ... ... ... ... ... ... ... ... ... ... ... ... ... ... ... ... ... ...  [360]
#AY877426    ... ... ... ... ... ... ... ... ... ... ... ... ... ... ... ... ... ... ... ...  [360]
#AY877428    ... ... ... ... ... ... ... ... ... ... ... ... ... ... ... ... ... ... ... ...  [360]
#AY877429    ... ... ... ... ... ... ... ... ... ... ... ... ... ... ... ... ... ... ... ...  [360]
#AY877427    ... ... ... ... ... ... ... ... ... ... ... ... ... ... ... ... ... ... ... ...  [360]
#AF417713    ... ... ... ... ... ... ... ... ... ... ... ... ... ... ... ... ... ... ... ...  [360]
#KR817728    ... ... ... ... ... ... ... ... ... ... ... ... ... ... ... ... ... ... ... ...  [360]

#PQ326383    GCT CAT TTT CAT TAT GTC TTA TCA ATA GGA GCA GTA TTT GCT ATT ATA GCA GGA TTT ATT  [420]
#PQ326384    ..C ... ... ... ... ... ... ... ... ... ... ... ... ... ... ... ... ... ... ...  [420]
#PQ326385    ... ... ... ... ... ... ... ... ... ... ... ... ... ... ... ... ... ... ... ...  [420]
#PQ326386    ... ... ... ... ... ... ... ... ... ... ... ... ... ... ... ... ... ... ... ...  [420]
#PQ326387    ... ... ... ... ... ... ... ... ... ... ... ... ... ... ... ... ... ... ... ...  [420]
#PQ326388    ..C ... ... ... ... ... ... ... ... ... ... ... ... ... ... ... ... ... ... ...  [420]
#PQ326389    ... ... ... ... ... ... ... ... ... ... ... ... ... ... ... ... ... ... ... ...  [420]
#PQ326390    ... ... ... ... ... ... ... ... ... ... ... ... ... ... ... ... ... ... ... ...  [420]
#PQ326391    ... ... ... ... ... ... ... ... ... ... ... ... ... ... ... ... ... ... ... ...  [420]
#MW012492    ... ... ... ... ... ... ... ... ... ... ... ... ... ... ... ... ... ... ... ...  [420]
#MZ269698    ... ... ... ... ... ... ... ... ... ... ... ... ... ... ... ... ... ... ... ...  [420]
#MZ269699    ... ... ... ... ... ... ... ... ... ... ... ... ... ... ... ... ... ... ... ...  [420]
#MZ269700    ... ... ... ... ... ... ... ... ... ... ... ... ... ... ... ... ... ... ... ...  [420]
#MZ269701    ... ... ... ... ... ... ... ... ... ... ... ... ... ... ... ... ... ... ... ...  [420]
#MZ269702    ... ... ... ... ... ... ... ... ... ... ... ... ... ... ... ... ... ... ... ...  [420]
#MZ269703    ... ... ... ... ... ... ... ... ... ... ... ... ... ... ... ... ... ... ... ...  [420]
#MZ269704    ... ... ... ... ... ... ... ... ... ... ... ... ... ... ... ... ... ... ... ...  [420]
#MZ269705    ... ... ... ... ... ... ... ... ... ... ... ... ... ... ... ... ... ... ... ...  [420]
#KT899888    ... ... ... ... ... ... ... ... ... ... ... ... ... ... ... ... ... ... ... ...  [420]
#AY877426    ... ... ... ... ... ... ... ... ... ... ... ... ... ... ... ... ... ... ... ...  [420]
#AY877428    ... ... ... ... ... ... ... ... ... ... ... ... ... ... ... ... ... ... ... ...  [420]
#AY877429    ... ... ... ... ... ... ... ... ... ... ... ... ... ... ... ... ... ... ... ...  [420]
#AY877427    ... ... ... ... ... ... ... ... ... ... ... ... ... ... ... ... ... ... ... ...  [420]
#AF417713    ... ... ... ... ... ... ... ... ... ... ... ... ... ... ... ... ... ... ... ...  [420]
#KR817728    ... ... ... ... ... ... ... ... ... ... ... ... ... ... ... ... ... ... ... ...  [420]

#PQ326383    CAC TGA TAC CCT TTA TTA ACA GGA TTA ACT ATA AAC CCT ACA TGA TTA AAA ATC CAA TTT  [480]
#PQ326384    ... ... ... ... ... ... ... ... ... ... ... ... ... ... ... ... ... ... ... ...  [480]
#PQ326385    ... ... ... ... ... ... ... ... ... ... ... ... ... ... ... ... ... ... ... ...  [480]
#PQ326386    ... ... ... ... ... ... ... ... ... ... ... ... ... ... ... ... ... ... ... ...  [480]
#PQ326387    ... ... ... ... ... ... ... ... ... ... ... ... ... ... ... ... ... ... ... ...  [480]
#PQ326388    ... ... ... ... ... ... ... ... ... ... ... ... ... ... ... ... ... ... ... ...  [480]
#PQ326389    ... ... ... ... ... ... ... ... ... ... ... ... ... ... ... ... ... ... ... ...  [480]
#PQ326390    ... ... ... ... ... ... ... ... ... ... ... ... ... ... ... ... ... ... ... ...  [480]
#PQ326391    ... ... ... ... ... ... ... ... ... ... ... ... ... ... ... ... ... ... ... ...  [480]
#MW012492    ... ... ... ... ... ... ... ... ... ... ... ... ... ... ... ... ... ... ... ...  [480]
#MZ269698    ... ... ... ... ... ... ... ... ... ... ... ... ... ... ... ... ... ... ... ...  [480]
#MZ269699    ... ... ... ... ... ... ... ... ... ... ... ... ... ... ... ... ... ... ... ...  [480]
#MZ269700    ... ... ... ... ... ... ... ... ... ... ... ... ... ... ... ... ... ... ... ...  [480]
#MZ269701    ... ... ... ... ... ... ... ... ... ... ... ... ... ... ... ... ... ... ... ...  [480]
#MZ269702    ... ... ... ... ... ... ... ... ... ... ... ... ... ... ... ... ... ... ... ...  [480]
#MZ269703    ... ... ... ... ... ... ... ... ... ... ... ... ... ... ... ... ... ... ... ...  [480]
```

```
#MZ269704     ... ... ... ... ... ... ... ... ... ... ... ... ... ... ... ... ... ... ... ...  [480]
#MZ269705     ... ... ... ... ... ... ... ... ... ... ... ... ... ... ... ... ... ... ... ...  [480]
#KT899888     ... ... ... ... ... ... ... ... ... ... ... ... ... ... ... ... ... ... ... ...  [480]
#AY877426     ... ... ... ... ... ... ... ... ... ... ... ... ... ... ... ... ... ... ... ...  [480]
#AY877428     ... ... ... ... ... ... ... ... ... ... ... ... ... ... ... ... ... ... ... ...  [480]
#AY877429     ... ... ... ... ... ... ... ... ... ... ... ... ... ... ... ... ... ... ... ...  [480]
#AY877427     ... ... ... ... ... ... ... ... ... ... ... ... ... ... ... ... ... ... ... ...  [480]
#AF417713     ... ... ... ... ... ... ... ... ... ... ... ... ... ... ... ... ... ... ... ...  [480]
#KR817728     ... ... ... ... ... ... ... ... ... ... ... ... ... ... ... ... ... ... ... ...  [480]

#PQ326383     TCT ATT ATA TTT ATT GGA GTA AAT TTA ACA TTT TTC CCG CAA CAT TTT CTA GGA TTA GCC  [540]
#PQ326384     ... ... ... ... ... ... ... ... ... ... ... ... ... ... ... ... ... ... ... ...  [540]
#PQ326385     ... ... ... ... ... ... ... ... ... ... ... ... ... ... ... ... ... ... ... ...  [540]
#PQ326386     ... ... ... ... ... ... ... ... ... ... ... ... ... ... ... ... ... ... ... ...  [540]
#PQ326387     ... ... ... ... ... ... ... ... ... ... ... ... ... ... ... ... ... ... ... ...  [540]
#PQ326388     ... ... ... ... ... ... ... ... ... ... ... ... ... ... ... ... ... ... ... ...  [540]
#PQ326389     ... ... ... ... ... ... ... ... ... ... ... ... ... ... ... ... ... ... ... ...  [540]
#PQ326390     ... ... ... ... ... ... ... ... ... ... ... ... ... ... ... ... ... ... ... ...  [540]
#PQ326391     ... ... ... ... ... ... ... ... ... ... ... ... ... ... ... ... ... ... ... ...  [540]
#MW012492     ... ... ... ... ... ... ... ... ... ... ... ... ... ... ... ... ... ... ... ...  [540]
#MZ269698     ... ... ... ... ... ... ... ... ... ... ... ... ... ... ... ... ... ... ... ...  [540]
#MZ269699     ... ... ... ... ... ... ... ... ... ... ... ... ... ... ... ... ... ... ... ...  [540]
#MZ269700     ... ... ... ... ... ... ... ... ... ... ... ... ... ... ... ... ... ... ... ...  [540]
#MZ269701     ... ... ... ... ... ... ... ... ... ... ... ... ... ... ... ... ... ... ... ...  [540]
#MZ269702     ... ... ... ... ... ... ... ... ... ... ... ... ... ... ... ... ... ... ... ...  [540]
#MZ269703     ... ... ... ... ... ... ... ... ... ... ... ... ... ... ... ... ... ... ... ...  [540]
#MZ269704     ... ... ... ... ... ... ... ... ... ... ... ... ... ... ... ... ... ... ... ...  [540]
#MZ269705     ... ... ... ... ... ... ... ... ... ... ... ... ... ... ... ... ... ... ... ...  [540]
#KT899888     ... ... ... ... ... ... ... ... ... ... ... ... ... ... ... ... ... ... ... ...  [540]
#AY877426     ... ... ... ... ... ... ... ... ... ... ... ... ..A ... ... ... ... ... ... ...  [540]
#AY877428     ... ... ... ... ... ... ... ... ... ... ... ... ..A ... ... ... ... ... ... ...  [540]
#AY877429     ... ... ... ... ... ... ... ... ... ... ... ... ..A ... ... ... ... ... ... ...  [540]
#AY877427     ... ... ... ... ... ... ... ... ... ... ... ... ..A ... ... ... ... ... ... ...  [540]
#AF417713     ... ... ... ... ... ... ... ... ... ... ... ... ... ... ... ... ... ... ... ...  [540]
#KR817728     ... ... ... ... ... ... ... ... ... ... ... ... ..A ... ... ... ... ... ... ...  [540]

#PQ326383     GGA ATA CCT CGA CGA TAT TCA GAT TTT CCA GAT AGT TAT CTA TCT TGA AAT ATT ATT TCT  [600]
#PQ326384     ... ... ... ... ... ... ... ... ... ... ... ... ... ... ... ... ... ... ... ...  [600]
#PQ326385     ... ... ... ... ... ... ... ... ... ... ... ... ... ... ... ... ... ... ... ...  [600]
#PQ326386     ... ... ... ... ... ... ... ... ... ... ... ... ... ... ... ... ... ... ... ...  [600]
#PQ326387     ... ... ... ... ... ... ... ... ... ... ... ... ... ... ... ... ... ... ... ...  [600]
#PQ326388     ... ... ... ... ... ... ... ... ... ... ... ... ... ... ... ... ... ... ... ...  [600]
#PQ326389     ... ... ... ... ... ... ... ... ... ... ... ... ... ... ... ... ... ... ... ...  [600]
#PQ326390     ... ... ... ... ... ... ... ... ... ... ... ... ... ... ... ... ... ... ... ...  [600]
#PQ326391     ... ... ... ... ... ... ... ... ... ... ... ... ... ... ... ... ... ... ... ...  [600]
#MW012492     ... ... ... ... ... ... ... ... ... ... ... ... ... ... ... ... ... ... ... ...  [600]
#MZ269698     ... ... ... ... ... ... ... ... ... ... ... ... ... ... ... ... ... ... ... ...  [600]
#MZ269699     ... ... ... ... ... ... ... ... ... ... ... ... ... ... ... ... ... ... ... ...  [600]
#MZ269700     ... ... ... ... ... ... ... ... ... ... ... ... ... ... ... ... ... ... ... ...  [600]
#MZ269701     ... ... ... ... ... ... ... ... ... ... ... ... ... ... ... ... ... ... ... ...  [600]
#MZ269702     ... ... ... ... ... ... ... ... ... ... ... ... ... ... ... ... ... ... ... ...  [600]
#MZ269703     ... ... ... ... ... ... ... ... ... ... ... ... ... ... ... ... ... ... ... ...  [600]
#MZ269704     ... ... ... ... ... ... ... ... ... ... ... ... ... ... ... ... ... ... ... ...  [600]
#MZ269705     ... ... ... ... ... ... ... ... ... ... ... ... ... ... ... ... ... ... ... ...  [600]
#KT899888     ... ... ... ... ... ... ... ... ... ... ... ... ... ... ... ... ... ... ... ...  [600]
#AY877426     ... ... ... ... ... ..C ... ... ... ... ... ... ... ... ... ... ... ... ... ...  [600]
#AY877428     ... ... ... ... ... ..C ... ... ... ... ... ... ... ... ... ... ... ... ... ...  [600]
#AY877429     ... ... ... ... ... ..C ... ... ... ... ... ... ... ... ... ... ... ... ... ...  [600]
#AY877427     ... ... ... ... ... ..C ... ... ... ... ... ... ... ... ... ... ... ... ... ...  [600]
#AF417713     ... ... ... ... ... ... ... ... ... ... ... ... ... ... ... ... ... ... ... ...  [600]
#KR817728     ... ... ... ... ... ..C ... ... ... ... ... ... ... ... ... ... ... ... ... ...  [600]

#PQ326383     TCA TTA GGA AGT ACA ATC TCA TTA TTT GCT ATT TTG TAT TTT TTA TTT ATT ATT TGA GAA  [660]
#PQ326384     ... ... ... ... ... ... ... ... ... ... ... ... ... ... ... ... ... ... ... ...  [660]
#PQ326385     ... C.. ... ... ... ... ... ... ... ... ... ... ... ... ... ... ... ... ... ...  [660]
#PQ326386     ... ... ... ... ... ... ... ... ... ... ... ... ... ... ... ... ... ... ... ...  [660]
```

```
#PQ326387    ... ... ... ... ... ... ... ... ... ... ... ... ... ... ... ... ... ... ... ...  [660]
#PQ326388    ... ... ... ... ... ... ... ... ... ... ... ... ... ... ... ... ... ... ... ...  [660]
#PQ326389    ... C.. ... ... ... ... ... ... ... ... ... ... ... ... ... ... ... ... ... ...  [660]
#PQ326390    ... ... ... ... ... ... ... ... ... ... ... ... ... ... ... ... ... ... ... ...  [660]
#PQ326391    ... ... ... ... ... ... ... ... ... ... ... ... ... ... ... ... ... ... ... ...  [660]
#MW012492    ... ... ... ... ... ... ... ... ... ... ... ... ... ... ... ... ... ... ... ...  [660]
#MZ269698    ... ... ... ... ... ... ... ... ... ... ... ... ... ... ... ... ... ... ... ...  [660]
#MZ269699    ... ... ... ... ... ... ... ... ... ... ... ... ... ... ... ... ... ... ... ...  [660]
#MZ269700    ... ... ... ... ... ... ... ... ... ... ... ... ... ... ... ... ... ... ... ...  [660]
#MZ269701    ... ... ... ... ... ... ... ... ... ... ... ... ... ... ... ... ... ... ... ...  [660]
#MZ269702    ... ... ... ... ... ... ... ... ... ... ... ... ... ... ... ... ... ... ... ...  [660]
#MZ269703    ... ... ... ... ... ... ... ... ... ... ... ... ... ... ... ... ... ... ... ...  [660]
#MZ269704    ... ... ... ... ... ... ... ... ... ... ... ... ... ... ... ... ... ... ... ...  [660]
#MZ269705    ... ... ... ... ... ... ... ... ... ... ... ... ... ... ... ... ... ... ... ...  [660]
#KT899888    ... ... ... ... ... ... ... ... ... ... ... ... ... ... ... ... ... ... ... ...  [660]
#AY877426    ... ... ... ... ... ... ... ... ... ... ... ..A ... ... ... ... ... ... ... ...  [660]
#AY877428    ... ... ... ... ... ... ... ... ... ... ... ..A ... ... ... ... ... ... ... ...  [660]
#AY877429    ... ... ... ... ... ... ... ... ... ... ... ..A ... ... ... ... ... ... ... ...  [660]
#AY877427    ... ... ... ... ... ... ... ... ... ... ... ..A ... ... ... ... ... ... ... ...  [660]
#AF417713    ... ... ... ... ... ... ... ... ... ... ... ... ... ... ... ... ... ... ... ...  [660]
#KR817728    ... ... ... ... ... ... ... ... ... ... ... ..A ... ... ... ... ... ... ... ...  [660]

#PQ326383    AGT ATA ATT ACA CAA CGT ACG CCT AGT TTT CCT ATA CAA TTG TCT TCA TCA ATT GAA TGA  [720]
#PQ326384    ... ... ... ... ... ... ..A ... ... ... ... ... ... ... ... ... ... ... ... ...  [720]
#PQ326385    ... ... ... ... ... ... ..A ... ... ... ... ... ... ... ... ... ... ... ... ...  [720]
#PQ326386    ... ... ... ... ... ... ... ... ... ... ... ... ... ... ... ... ... ... ... ...  [720]
#PQ326387    ... ... ... ... ... ... ... ... ... ... ... ... ... ... ... ... ... ... ... ...  [720]
#PQ326388    ... ... ... ... ... ... ..A ... ... ... ... ... ... ... ... ... ... ... ... ...  [720]
#PQ326389    ... ... ... ... ... ... ..A ... ... ... ... ... ... ... ... ... ... ... ... ...  [720]
#PQ326390    ... ... ... ... ... ... ... ... ... ... ... ... ... ... ... ... ... ... ... ...  [720]
#PQ326391    ... ... ... ... ... ... ... ... ... ... ... ... ... ... ... ... ... ... ... ...  [720]
#MW012492    ... ... ... ... ... ... ..A ... ... ... ... ... ... ... ... ... ... ... ... ...  [720]
#MZ269698    ... ... ... ... ... ... ..A ... ... ... ... ... ... ... ... ... ... ... ... ...  [720]
#MZ269699    ... ... ... ... ... ... ..A ... ... ... ... ... ... ... ... ... ... ... ... ...  [720]
#MZ269700    ... ... ... ... ... ... ..A ... ... ... ... ... ... ... ... ... ... ... ... ...  [720]
#MZ269701    ... ... ... ... ... ... ..A ... ... ... ... ... ... ... ... ... ... ... ... ...  [720]
#MZ269702    ... ... ... ... ... ... ..A ... ... ... ... ... ... ... ... ... ... ... ... ...  [720]
#MZ269703    ... ... ... ... ... ... ..A ... ... ... ... ... ... ... ... ... ... ... ... ...  [720]
#MZ269704    ... ... ... ... ... ... ..A ... ... ... ... ... ... ... ... ... ... ... ... ...  [720]
#MZ269705    ... ... ... ... ... ... ..A ... ... ... ... ... ... ... ... ... ... ... ... ...  [720]
#KT899888    ... ... ... ... ... ... ..A ... ... ... ... ... ... ... ... ... ... ... ... ...  [720]
#AY877426    ... ... ... ... ... ... ..A ... ... ... ... ... ... ..A ... ... ... ... ... ...  [720]
#AY877428    ... ... ... ... ... ... ..A ... ... ... ... ... ... ..A ... ... ... ... ... ...  [720]
#AY877429    ... ... ... ... ... ... ..A ... ... ... ... ... ... ..A ... ... ... ... ... ...  [720]
#AY877427    ... ... ... ... ... ... ..A ... ... ... ... ... ... ..A ... ... ... ... ... ...  [720]
#AF417713    ... ... ... ... ... ... ..A ... ... ... ... ... ... ... ... ... ... ... ... ...  [720]
#KR817728    ... ... ... ... ... ... ..A ... ... ... ... ... ... ..A ... ... ... ... ... ...  [720]

#PQ326383    TAT CAT ACC CTT CCC CCA GCT GAG CAT ACA TAT GCA GA [758]
#PQ326384    ... ... ... ... ... ... ... ... ... ... ... ... .. [758]
#PQ326385    ... ... ... ... ... ... ... ... ... ... ... ... .. [758]
#PQ326386    ... ... ... ... ... ... ... ... ... ... ... ... .. [758]
#PQ326387    ... ... ... ... ... ... ... ... ... ... ... ... .. [758]
#PQ326388    ... ... ... ... ... ... ... ... ... ... ... ... .. [758]
#PQ326389    ... ... ... ... ... ... ... ... ... ... ... ... .. [758]
#PQ326390    ... ... ... ... ... ... ... ... ... ... ... ... .. [758]
#PQ326391    ... ... ... ... ... ... ... ... ... ... ... ... .. [758]
#MW012492    ... ... ... ... ... ... ... ... ... ... ... ... .. [758]
#MZ269698    ... ... ... ... ... ... ... ... ... ... ... ... .. [758]
#MZ269699    ... ... ... ... ... ... ... ... ... ... ... ... .. [758]
#MZ269700    ... ... ... ... ... ... ... ... ... ... ... ... .. [758]
#MZ269701    ... ... ... ... ... ... ... ... ... ... ... ... .. [758]
#MZ269702    ... ... ... ... ... ... ... ... ... ... ... ... .. [758]
#MZ269703    ... ... ... ... ... ... ... ... ... ... ... ... .. [758]
#MZ269704    ... ... ... ... ... ... ... ... ... ... ... ... .. [758]
#MZ269705    ... ... ... ... ... ... ... ... ... ... ... ... .. [758]
```

```
#KT899888     ... ... ... ... ... ... ... ... ... ... ... ... .. [758]
#AY877426     ... ... ..T ... ... ... ... ... ... ... ... ... .. [758]
#AY877428     ... ... ..T ... ... ... ... ... ... ... ... ... .. [758]
#AY877429     ... ... ..T ... ... ... ... ... ... ... ... ... .. [758]
#AY877427     ... ... ..T ... ... ... ... ... ... ... ... ... .. [758]
#AF417713     ... ... ... ... ... ... ... ... ... ... ... ... .. [758]
#KR817728     ... ... ..T ... ... ... ... ... ... ... ... ... .. [758]
```
