# Supplementary material for: Phylogeographic Patterns and Genetic Diversity of Anopheles stephensi: Implications for Global Malaria Transmission
Source: Trop Med Infect Dis. 2025 Apr 16;10(4):109. doi: 10.3390/tropicalmed10040109 (PMC12031451; doi:10.3390/tropicalmed10040109)
Supplement: Supplementary file 1 [file tropicalmed-10-00109-s001.zip › Supplementary File S3 COII Sequences.pdf]

```
#PQ431427     TTC TTC ACC TTT AAT AGA ACA ATT AAA TTT TTT TCA TGA TCA TAC ACT TCT TAT TTT AAC  [ 60]
#PQ431428     ... ... ... ... ... ... ... ... ... ... ... ... ... ... ... ... ... ... ... ...  [ 60]
#PQ431429     ... ... ... ... ... ... ... ... ... ... ... ... ... ... ... ... ... ... ... ...  [ 60]
#PQ431430     ... ... ... ... ... ... ... ... ... ... ... ... ... ... ... ... ... ... ... ...  [ 60]
#PQ431431     ... ... ... ... ... ... ... ... ... ... ... ... ... ... ... ... ... ... ... ...  [ 60]
#MZ420723     ... ... ... ... ... ... ... ... ... ... ... ... ... ... ... ... ... ... ... ...  [ 60]
#MZ420724     ... ... ... ... ... ... ... ... ... ... ... ... ... ... ... ... ... ... ... ...  [ 60]
#MZ420725     ... ... ... ... ... ... ... ... ... ... ... ... ... ... ... ... ... ... ... ...  [ 60]
#MZ420726     ... ... ... ... ... ... ... ... ... ... ... ... ... ... ... ... ... ... ... ...  [ 60]
#MZ420727     ... ... ... ... ... ... ... ... ... ... ... ... ... ... ... ... ... ... ... ...  [ 60]
#MZ420728     ... ... ... ... ... ... ... ... ... ... ... ... ... ... ... ... ... ... ... ...  [ 60]
#MZ420729     ... ... ... ... ... ... ... ... ... ... ... ... ... ... ... ... ... ... ... ...  [ 60]
#MZ420730     ... ... ... ... ... ... ... ... ... ... ... ... ... ... ... ... ... ... ... ...  [ 60]
#MW431057     ... ... ... ... ... ... ... ... ... ... ... ... ... ... ... ... ... ... ... ...  [ 60]
#KT899888     ... ... ... ... ... ... ... ... ... ... ... ... ... ... ... ... ... ... ... ...  [ 60]
#AF425844     ... ... ... ... ... ... ... ... ... ... ... ... ... ... ... ... ... ... ... ...  [ 60]
#DQ022847     ... ... ... ... ... ... ... ... ... ... ... ... ... ... ... ... ... ... ... ...  [ 60]
#DQ022846     ... ... ... ... ... ... ... ... ... ... ... ... ... ... ... ... ... ... ... ...  [ 60]
#DQ022845     ... ... ... ... ... ... ... ... ... ... ... ... ... ... ... ... ... ... ... ...  [ 60]
#DQ026675     ... ... ... ... ... ... ... ... ... ... ... ... ... ... ... ... ... ... ... ...  [ 60]
#AY883832     ... ... ... ... ... ... ... ... ... ... ... ... ... ... ... ... ... ... ... ...  [ 60]
#AY883830     ... ... ... ... ... ... ... ... ... ... ... ... ... ... ... ... ... ... ... ...  [ 60]
#AY883837     ... ... ... ... ... ... ... ... ... ... ... ... ... ... ... ... ... ... ... ...  [ 60]
#AY883836     ... ... ... ... ... ... ... ... ... ... ... ... ... ... ... ... ... ... ... ...  [ 60]
#AY883835     ... ... ... ... ... ... ... ... ... ... ... ... ... ... ... ... ... ... ... ...  [ 60]
#AY883834     ... ... ... ... ... ... ... ... ... ... ... ... ... ... ... ... ... ... ... ...  [ 60]
#AY883833     ... ... ... ... ... ... ... ... ... ... ... ... ... ... ... ... ... ... ... ...  [ 60]
#AY883831     ... ... ... ... ... ... ... ... ... ... ... ... ... ... ... ... ... ... ... ...  [ 60]
#KY863454     ... ... ... ... ... ... ... ... ... ... ... ... ... ... ... ... ... ... ... ...  [ 60]
#EF208912     ... ... .G. ... G.. ... ... ... ... ... ... ... ... ... ... ... ... ... ... ...  [ 60]
#FJ526438     ... ... ... ... ... ... ... ... ... ... ... ... ... ... ... ... ... ... ... ...  [ 60]
#FJ526437     ... ... ... ... ... ... ... ... ... ... ... ... ... ... ... ... ... ... ... ...  [ 60]
#AF417749     ... ... ... ... ... ... ... ... ... ... ... ... ... ... ... ... ... ... ... ...  [ 60]
#JX139608     ... ... ... ... ... ... ... ... ... ... ... ... ... ... ... ... ... ... ... ...  [ 60]

#PQ431427     TAT AAT TAC AAT TTT AGT AGG ATA TAT TAT AGG AAT ATT AAT ATT TAA TAA ATT TAC TAA  [120]
#PQ431428     ... ... ... ... ... ... ... ... ... ... ... ... ... ... ... ... ... ... ... ...  [120]
#PQ431429     ... ... ... ... ... ... ... ... ... ... ... ... ... ... ... ... ... ... ... ...  [120]
#PQ431430     ... ... ... ... ... ... ... ... ... ... ... ... ... ... ... ... ... ... ... ...  [120]
#PQ431431     ... ... ... ... ... ... ... ... ... ... ... ... ... ... ... ... ... ... ... ...  [120]
#MZ420723     ... ... ... ... ... ... ... ... ... ... ... ... ... ... ... ... ... ... ... ...  [120]
#MZ420724     ... ... ... ... ... ... ... ... ... ... ... ... ... ... ... ... ... ... ... ...  [120]
#MZ420725     ... ... ... ... ... ... ... ... ... ... ... ... ... ... ... ... ... ... ... ...  [120]
#MZ420726     ... ... ... ... ... ... ... ... ... ... ... ... ... ... ... ... ... ... ... ...  [120]
#MZ420727     ... ... ... ... ... ... ... ... ... ... ... ... ... ... ... ... ... ... ... ...  [120]
#MZ420728     ... ... ... ... ... ... ... ... ... ... ... ... ... ... ... ... ... ... ... ...  [120]
#MZ420729     ... ... ... ... ... ... ... ... ... ... ... ... ... ... ... ... ... ... ... ...  [120]
#MZ420730     ... ... ... ... ... ... ... ... ... ... ... ... ... ... ... ... ... ... ... ...  [120]
#MW431057     ... ... ... ... ... ... ... ... ... ... ... ... ... ... ... ... ... ... ... ...  [120]
#KT899888     ... ... ... ... ... ... ... ... ... ... ... ... ... ... ... ... ... ... ... ...  [120]
#AF425844     ... ... ... ... ... ... ... ... ... ... ... ... ... ... ... ... ... ... ... ...  [120]
#DQ022847     ... ... ... ... ... ... ... ... ... ... ... ... ... ... ... ... ... ... ... ...  [120]
#DQ022846     ... ... ... ... ... ... ... ... ... ... ... ... ... ... ... ... ... ... ... ...  [120]
#DQ022845     ... ... ... ... ... ... ... ... ... ... ... ... ... ... ... ... ... ... ... ...  [120]
#DQ026675     ... ... ... ... ... ... ... ... ... ... ... ... ... ... ... ... ... ... ... ...  [120]
#AY883832     ... ... ... ... ... ... ... ... ... ... ... ... ... ... ... ... ... ... ... ...  [120]
#AY883830     ... ... ... ... ... ... ... ... ... ... ... ... ... ... ... ... ... ... ... ...  [120]
#AY883837     ... ... ... ... ... ... ... ... ... ... ... ... ... ... ... ... ... ... ... ...  [120]
#AY883836     ... ... ... ... ... ... ... ... ... ... ... ... ... ... ... ... ... ... ... ...  [120]
#AY883835     ... ... ... ... ... ... ... ... ... ... ... ... ... ... ... ... ... ... ... ...  [120]
#AY883834     ... ... ... ... ... ... ... ... ... ... ... ... ... ... ... ... ... ... ... ...  [120]
#AY883833     ... ... ... ... ... ... ... ... ... ... ... ... ... ... ... ... ... ... ... ...  [120]
#AY883831     ... ... ... ... ... ... ... ... ... ... ... ... ... ... ... ... ... ... ... ...  [120]
```

```
#KY863454     ... ... ... ... ... ... ... ... ... ... ... ... ... ... ... ... ... ... ... ...  [120]
#EF208912     ... ... ... ... ... ... ... ... ... ... ... ... ... ... ... ... ... ... ... ...  [120]
#FJ526438     ... ... ... ... ... ... ... ... ... ... ... ... ... ... ... ... ... ... ... ...  [120]
#FJ526437     ... ... ... ... ... ... ... ... ... ... ... ... ... ... ... ... ... ... ... ...  [120]
#AF417749     ... ... ... ... ... ... ... ... ... ... ... ... ... ... ... ... ... ... ... ...  [120]
#JX139608     ... ... ... ... ... ... ... ... ... ... ... ... ... ... ... ... ... ... ... ...  [120]

#PQ431427     TCG ATT TTT ACT TCA TGG ACA AAC AAT TGA AAT TAT TTG AAC AGT TCT ACC AGC AAT TAT  [180]
#PQ431428     ... ... ... ... ... ... ... ... ... ... ... ... ... ... ... ... ... ... ... ...  [180]
#PQ431429     ... ... ... ... ... ... ... ... ... ... ... ... ... ... ... ... ... ... ... ...  [180]
#PQ431430     ... ... ... ... ... ... ... ... ... ... ... ... ... ... ... ... ... ... ... ...  [180]
#PQ431431     ... ... ... ... ... ... ... ... ... ... ... ... ... ... ... ... ... ... ... ...  [180]
#MZ420723     ... ... ... ... ... ... ... ... ... ... ... ... ... ... ... ... ... ... ... ...  [180]
#MZ420724     ... ... ... ... ... ... ... ... ... ... ... ... ... ... ... ... ... ... ... ...  [180]
#MZ420725     ... ... ... ... ... ... ... ... ... ... ... ... ... ... ... ... ... ... ... ...  [180]
#MZ420726     ... ... ... ... ... ... ... ... ... ... ... ... ... ... ... ... ... ... ... ...  [180]
#MZ420727     ... ... ... ... ... ... ... ... ... ... ... ... ... ... ... ... ... ... ... ...  [180]
#MZ420728     ... ... ... ... ... ... ... ... ... ... ... ... ... ... ... ... ... ... ... ...  [180]
#MZ420729     ... ... ... ... ... ... ... ... ... ... ... ... ... ... ... ... ... ... ... ...  [180]
#MZ420730     ... ... ... ... ... ... ... ... ... ... ... ... ... ... ... ... ... ... ... ...  [180]
#MW431057     ... ... ... ... ... ... ... ... ... ... ... ... ... ... ... ... ... ... ... ...  [180]
#KT899888     ... ... ... ... ... ... ... ... ... ... ... ... ... ... ... ... ... ... ... ...  [180]
#AF425844     ... ... ... ... ... ... ... ... ... ... ... ... ... ... .A. ... ... ... ... ...  [180]
#DQ022847     ... ... ... ... ... ... ... ... ... ... ... ... ... ... ... ... ... ... ... ...  [180]
#DQ022846     ... ... ... ... ... ... ... ... ... ... ... ... ... ... ... ... ... ... ... ...  [180]
#DQ022845     ... ... ... ... ... ... ... ... ... ... ... ... ... ... ... .T. ... ... ... ...  [180]
#DQ026675     ... ... ... ... ... ... ... ... ... ... ... ... ... ... .A. ... ... ... ... ...  [180]
#AY883832     ... ... ... ... ... ... ... ... ... ... ... ... ... ... ... .T. ... ... ... ...  [180]
#AY883830     ... ... ... ... ... ... ... ... ... ... ... ... ... ... ... .T. ... ... ... ...  [180]
#AY883837     ... ... ... ... ... ... ... ... ... ... ... ... ... ... ... .T. ... ... ... ...  [180]
#AY883836     ... ... ... ... ... ... ... ... ... ... ... ... ... ... ... .T. ... ... ... ...  [180]
#AY883835     ... ... ... ... ... ... ... ... ... ... ... ... ... ... ... .T. ... ... ... ...  [180]
#AY883834     ... ... ... ... ... ... ... ... ... ... ... ... ... ... ... .T. ... ... ... ...  [180]
#AY883833     ... ... ... ... ... ... ... ... ... ... ... ... ... ... ... .T. ... ... ... ...  [180]
#AY883831     ... ... ... ... ... ... ... ... ... ... ... ... ... ... ... .T. ... ... ... ...  [180]
#KY863454     ... ... ... ... ... ... ... ... ... ... ... ... ... ... ... .T. ... ... ... ...  [180]
#EF208912     ... ... ... ... ... ... ... ... ... ... ... ... ... ... ... ... ... ... ... ...  [180]
#FJ526438     ... ... ... ... ... ... ... ... ... ... ... ... ... ... ... .T. ... ... ... ...  [180]
#FJ526437     ... ... ... ... ... ... ... ... ... ... ... ... ... ... ... ... ... ... ... ...  [180]
#AF417749     ... ... ... ... ... ... ... ... ... ... ... ... ... ... .A. ... ... ... ... ...  [180]
#JX139608     ... ... ... ... ... ... ... ... ... ... ... ... ... ... ... ... ... ... ... ...  [180]

#PQ431427     TTT AAT ATT TAT TGC CTT TCC ATC TTT ACG ATT ATT ATA TTT AAT AGA TGA AAT TAA TAC  [240]
#PQ431428     ... ... ... ... ... ... ... ... ... ... ... ... ... ... ... ... ... ... ... ...  [240]
#PQ431429     ... ... ... ... ... ... ... ... ... ... ... ... ... ... ... ... ... ... ... ...  [240]
#PQ431430     ... ... ... ... ... ... ... ... ... ... ... ... ... ... ... ... ... ... ... ...  [240]
#PQ431431     ... ... ... ... ... ... ... ... ... ... ... ... ... ... ... ... ... ... ... ...  [240]
#MZ420723     ... ... ... ... ... ... ... ... ... ... ... ... ... ... ... ... ... ... ... ...  [240]
#MZ420724     ... ... ... ... ... ... ... ... ... ... ... ... ... ... ... ... ... ... ... ...  [240]
#MZ420725     ... ... ... ... ... ... ... ... ... ... ... ... ... ... ... ... ... ... ... ...  [240]
#MZ420726     ... ... ... ... ... ... ... ... ... ... ... ... ... ... ... ... ... ... ... ...  [240]
#MZ420727     ... ... ... ... ... ... ... ... ... ... ... ... ... ... ... ... ... ... ... ...  [240]
#MZ420728     ... ... ... ... ... ... ... ... ... ... ... ... ... ... ... ... ... ... ... ...  [240]
#MZ420729     ... ... ... ... ... ... ... ... ... ... ... ... ... ... ... ... ... ... ... ...  [240]
#MZ420730     ... ... ... ... ... ... ... ... ... ... ... ... ... ... ... ... ... ... ... ...  [240]
#MW431057     ... ... ... ... ..T ... ... ... ... ... ... ... ... ... ... ... ... ... ... ...  [240]
#KT899888     ... ... ... ... ... ... ... ... ... ... ... ... ... ... ... ... ... ... ... ...  [240]
#AF425844     ... ... ... ... ... ... ... ... ... ... ... ... ... ... ... ... ... ... ... ...  [240]
#DQ022847     ... ... ... ... ... ... ... ... ... ... ... ... ... ... ... ... ... ... ... ...  [240]
#DQ022846     ... ... ... ... ... ... ... ... ... ... ... ... ... ... ... ... ... ... ... ...  [240]
#DQ022845     ... ... ... ... ... ... ... ... ... ... ... ... ... ... ... ... ... ... ... ...  [240]
#DQ026675     ... ... ... ... ... ... ... ... ... ... ... ... ... ... ... ... ... ... ... ...  [240]
#AY883832     ... ... ... ... ... ... ... ... ... ... ... ... ... ... ... ... ... ... ... ...  [240]
```

```
#AY883830     ... ... ... ... ... ... ... ... ... ... ... ... ... ... ... ... ... ... ... ...   [240]
#AY883837     ... ... ... ... ... ... ... ... ... ... ... ... ... ... ... ... ... ... ... ...   [240]
#AY883836     ... ... ... ... ... ... ... ... ... ... ... ... ... ... ... ... ... ... ... ...   [240]
#AY883835     ... ... ... ... ... ... ... ... ... ... ... ... ... ... ... ... ... ... ... ...   [240]
#AY883834     ... ... ... ... ... ... ... ... ... ... ... ... ... ... ... ... ... ... ... ...   [240]
#AY883833     ... ... ... ... ... ... ... ... ... ... ... ... ... ... ... ... ... ... ... ...   [240]
#AY883831     ... ... ... ... ... ... ... ... ... ... ... ... ... ... ... ... ... ... ... ...   [240]
#KY863454     ... ... ... ... ... ... ... ... ... ... ... ... ... ... ... ... ... ... ... ...   [240]
#EF208912     ... ... ... ... ... ... ... ... ... ... ... ... ... ... ... ... ... ... ... ...   [240]
#FJ526438     ... ... ... ... ... ... ... ... ... ... ... ... ... ... ... ... ... ... ... ...   [240]
#FJ526437     ... ... ... ... ... ... ... ... ... ... ... ... ... ... ... ... ... ... ... ...   [240]
#AF417749     ... ... ... ... ... ... ... ... ... ... ... ... ... ... ... ... ... ... ... ...   [240]
#JX139608     ... ... ... ... ... ... ... ... ... ... ... ... ... ... ... ... ... ... ... ...   [240]

#PQ431427     TCC ATC TAT TAC CTT AAA ATC AAT TGG ACA TCA ATG ATA CTG AAG ATA TGA ATA TTC TGA   [300]
#PQ431428     ... ... ... ... ... ... ... ... ... ... ... G.. ... ... ... ... ... ... ... ...   [300]
#PQ431429     ... ... ... ... ... ... ... ... ... ... ... G.. ... ... ... ... ... ... ... ...   [300]
#PQ431430     ... ... ... ... ... ... ... ... ... ... ... ... ... ... ... ... ... ... ... ...   [300]
#PQ431431     ... ... ... ... ... ... ... ... ... ... ... ... ... ... ... ... ... ... ... ...   [300]
#MZ420723     ... ... ... ... ... ... ... ... ... ... ... G.. ... ... ... ... ... ... ... ...   [300]
#MZ420724     ... ... ... ... ... ... ... ... ... ... ... G.. ... ... ... ... ... ... ... ...   [300]
#MZ420725     ... ... ... ... ... ... ... ... ... ... ... G.. ... ... ... ... ... ... ... ...   [300]
#MZ420726     ... ... ... ... ... ... ... ... ... ... ... G.. ... ... ... ... ... ... ... ...   [300]
#MZ420727     ... ... ... ... ... ... ... ... ... ... ... G.. ... ... ... ... ... ... ... ...   [300]
#MZ420728     ... ... ... ... ... ... ... ... ... ... ... G.. ... ... ... ... ... ... ... ...   [300]
#MZ420729     ... ... ... ... ... ... ... ... ... ... ... G.. ... ... ... ... ... ... ... ...   [300]
#MZ420730     ... ... ... ... ... ... ... ... ... ... ... G.. ... ... ... ... ... ... ... ...   [300]
#MW431057     ... ... ... ... ... ... ... ... ... ... ... G.. ... ... ... ... ... ... ... ...   [300]
#KT899888     ... ... ... ... ... ... ... ... ... ... ... G.. ... ... ... ... ... ... ... ...   [300]
#AF425844     ... ... ... ... ... ... ... ... ... ... ... G.. ... ... ... ... ... ... ... ...   [300]
#DQ022847     ... ... ... ... ... ... ... ... ... ... ... G.. ... ... ... ... ... ... ... ...   [300]
#DQ022846     ... ... ... ... ... ... ... ... ... ... ... G.. ... ... ... ... ... ... ... ...   [300]
#DQ022845     ... ... ... ... T.. ... ... ... ... ... ... G.. ... ... ... ... ... ... ... ...   [300]
#DQ026675     ... ... ... ... ... ... ... ... ... ... ... G.. ... ... ... ... ... ... ... ...   [300]
#AY883832     ... ... ... ... T.. ... ... ... ... ... ... G.. ... ... ... ... ... ... ... ...   [300]
#AY883830     ... ... ... ... T.. ... ... ... ... ... ... G.. ... ... ... ... ... ... ... ...   [300]
#AY883837     ... ... ... ... T.. ... ... ... ... ... ... G.. ... ... ... ... ... ... ... ...   [300]
#AY883836     ... ... ... ... T.. ... ... ... ... ... ... G.. ... ... ... ... ... ... ... ...   [300]
#AY883835     ... ... ... ... T.. ... ... ... ... ... ... G.. ... ... ... ... ... ... ... ...   [300]
#AY883834     ... ... ... ... T.. ... ... ... ... ... ... G.. ... ... ... ... ... ... ... ...   [300]
#AY883833     ... ... ... ... T.. ... ... ... ... ... ... G.. ... ... ... ... ... ... ... ...   [300]
#AY883831     ... ... ... ... T.. ... ... ... ... ... ... G.. ... ... ... ... ... ... ... ...   [300]
#KY863454     ... ... ... ... T.. ... ... ... ... ... ... G.. ... ... ... ... ... ... ... ...   [300]
#EF208912     ... ... ... ... ... ... ... ... ... ... ... G.. ... ... ... ... ... ... ... ...   [300]
#FJ526438     ... ... ... ... T.. ... ... ... ... ... ... G.. ... ... ... ... ... ... ... ...   [300]
#FJ526437     ... ... ... ... ... ... ... ... ... ... ... G.. ... ... ... ... ... ... ... ...   [300]
#AF417749     ... ... ... ... ... ... ... ... ... ... ... G.. ... ... ... ... ... ... ... ...   [300]
#JX139608     ... ... ... ... ... ... ... ... ... ... ... G.. ... ... ... ... ... ... ... ...   [300]

#PQ431427     TTT TTT AAA TTT AGA ATT TGA TTC ATA TAT AAT TCC TAC TAA TGA ATT AGA AAC AAA CGG   [360]
#PQ431428     ... ... ... ... ... ... ... ... ... ... ... ... ... ... ... ... ... ... ... ...   [360]
#PQ431429     ... ... ... ... ... ... ... ... ... ... ... ... ... ... ... ... ... ... ... ...   [360]
#PQ431430     ... ... ... ... ... ... ... ... ... ... ... ... ... ... ... ... ... ... ... ...   [360]
#PQ431431     ... ... ... ... ... ... ... ... ... ... ... ... ... ... ... ... ... ... ... ...   [360]
#MZ420723     ... ... ... ... ... ... ... ... ... ... ... ... ... ... ... ... ... ... ... ...   [360]
#MZ420724     ... ... ... ... ... ... ... ... ... ... ... ... ... ... ... ... ... ... ... ...   [360]
#MZ420725     ... ... ... ... ... ... ... ... ... ... ... ... ... ... ... ... ... ... ... ...   [360]
#MZ420726     ... ... ... ... ... ... ... ... ... ... ... ... ... ... ... ... ... ... ... ...   [360]
#MZ420727     ... ... ... ... ... ... ... ... ... ... ... ... ... ... ... ... ... ... ... ...   [360]
#MZ420728     ... ... ... ... ... ... ... ... ... ... ... ... ... ... ... ... ... ... ... ...   [360]
#MZ420729     ... ... ... ... ... ... ... ... ... ... ... ... ... ... ... ... ... ... ... ...   [360]
#MZ420730     ... ... ... ... ... ... ... ... ... ... ... ... ... ... ... ... ... ... ... ...   [360]
#MW431057     ... ... ... ... ... ... ... ... ... ... ... ... ... ... ... ... ... ... ... ...   [360]
```

```
#KT899888     ... ... ... ... ... ... ... ... ... ... ... ... ... ... ... ... ... ... ... ...  [360]
#AF425844     ... ... ... ... ... ... ... ... ... ... ... ... ... ... ... ... ... ... ... ...  [360]
#DQ022847     ... ... ... ... ... ... ... ... ... ... ... ... ... ... ... ... ... ... ... ...  [360]
#DQ022846     ... ... ... ... ... ... ... ... ... ... ... ... ... ... ... ... ... ... ... ...  [360]
#DQ022845     ... ... ... ... ... ... ... ... G.. ... ... ... ... ... ... ... ... ... ... ...  [360]
#DQ026675     ... ... ... ... ... ... ... ... ... ... ... ... ... ... ... ... ... ... ... ...  [360]
#AY883832     ... ... ... ... ... ... ... ... G.. ... ... ... ... ... ... ... ... ... ... ...  [360]
#AY883830     ... ... ... ... ... ... ... ... G.. ... ... ... ... ... ... ... ... ... ... ...  [360]
#AY883837     ... ... ... ... ... ... ... ... G.. ... ... ... ... ... ... ... ... ... ... ...  [360]
#AY883836     ... ... ... ... ... ... ... ... G.. ... ... ... ... ... ... ... ... ... ... ...  [360]
#AY883835     ... ... ... ... ... ... ... ... G.. ... ... ... ... ... ... ... ... ... ... ...  [360]
#AY883834     ... ... ... ... ... ... ... ... G.. ... ... ... ... ... ... ... ... ... ... ...  [360]
#AY883833     ... ... ... ... ... ... ... ... G.. ... ... ... ... ... ... ... ... ... ... ...  [360]
#AY883831     ... ... ... ... ... ... ... ... G.. ... ... ... ... ... ... ... ... ... ... ...  [360]
#KY863454     ... ... ... ... ... ... ... ... G.. ... ... ... ... ... ... ... ... ... ... ...  [360]
#EF208912     ... ... ... ... ... ... ... ... ... ... ... ... ... ... ... ... ... ... ... ...  [360]
#FJ526438     ... ... ... ... ... ... ... ... G.. ... ... ... ... ... ... ... ... ... ... ...  [360]
#FJ526437     ... ... ... ... ... ... ... ... ... ... ... ... ... ... ... ... ... ... ... ...  [360]
#AF417749     ... ... ... ... ... ... ... ... ... ... ... ... ... ... ... ... ... ... ... ...  [360]
#JX139608     ... ... ... ... ... ... ... ... ... ... ... ... ... ... ... ... ... ... ... ...  [360]

#PQ431427     ATT TCG ATT ATT AGA TGT TGA TAA TCG AGT TGT TTT ACC AAT AAA CAA TCA AAT TCG AAT  [420]
#PQ431428     ... ... ... ... ... ... ... ... ... ... ... ... ... ... ... ... ... ... ... ...  [420]
#PQ431429     ... ... ... ... ... ... ... ... ... ... ... ... ... ... ... ... ... ... ... ...  [420]
#PQ431430     ... ... ... ... ... ... ... ... ... ... ... ... ... ... ... ... ... ... ... ...  [420]
#PQ431431     ... ... ... ... ... ... ... ... ... ... ... ... ... ... ... ... ... ... ... ...  [420]
#MZ420723     ... ... ... ... ... ... ... ... ... ... ... ... ... ... ... ... ... ... ... ...  [420]
#MZ420724     ... ... ... ... ... ... ... ... ... ... ... ... ... ... ... ... ... ... ... ...  [420]
#MZ420725     ... ... ... ... ... ... ... ... ... ... ... ... ... ... ... ... ... ... ... ...  [420]
#MZ420726     ... ... ... ... ... ... ... ... ... ... ... ... ... ... ... ... ... ... ... ...  [420]
#MZ420727     ... ... ... ... ... ... ... ... ... ... ... ... ... ... ... ... ... ... ... ...  [420]
#MZ420728     ... ... ... ... ... ... ... ... ... ... ... ... ... ... ... ... ... ... ... ...  [420]
#MZ420729     ... ... ... ... ... ... ... ... ... ... ... ... ... ... ... ... ... ... ... ...  [420]
#MZ420730     ... ... ... ... ... ... ... ... ... ... ... ... ... ... ... ... ... ... ... ...  [420]
#MW431057     ... ... ... ... ... ... ... ... ... ... ... ... ... ... ... ... ... ... ... ...  [420]
#KT899888     ... ... ... ... ... ... ... ... ... ... ... ... ... ... ... ... ... ... ... ...  [420]
#AF425844     ... ... ... ... ... ... ... ... ... ... ... ... ... ... ... ... ... ... ... ...  [420]
#DQ022847     ... ... ... ... ... ... ... ... ... ... ... ... ... ... ... ... ... ... ... ...  [420]
#DQ022846     ... ... ... ... ... ... ... ... ... ... ... ... ... ... ... ... ... ... ... ...  [420]
#DQ022845     G.. ... ... G.. ... ... ... ... ... ... ... ... ... ... ... ... ... ... ... ...  [420]
#DQ026675     ... ... ... ... ... ... ... ... ... ... ... ... ... ... ... ... ... ... ... ...  [420]
#AY883832     G.. ... ... G.. ... ... ... ... ... ... ... ... ... ... ... ... ... ... ... ...  [420]
#AY883830     G.. ... ... G.. ... ... ... ... ... ... ... ... ... ... ... ... ... ... ... ...  [420]
#AY883837     G.. ... ... G.. ... ... ... ... ... ... ... ... ... ... ... ... ... ... ... ...  [420]
#AY883836     G.. ... ... G.. ... ... ... ... ... ... ... ... ... ... ... ... ... ... ... ...  [420]
#AY883835     G.. ... ... G.. ... ... ... ... ... ... ... ... ... ... ... G.. ... ... ... ...  [420]
#AY883834     G.. ... ... G.. ... ... ... ... ... ... ... ... ... ... ... ... ... ... ... ...  [420]
#AY883833     G.. ... ... G.. ... ... ... ... ... ... ... ... ... ... ... ... ... ... ... ...  [420]
#AY883831     G.. ... ... G.. ... ... ... ... ... ... ... ... ... ... ... ... ... ... ... ...  [420]
#KY863454     G.. ... ... G.. ... ... ... ... ... ... ... ... ... ... ... ... ... ... ... ...  [420]
#EF208912     ... ... ... ... ... ... ... ... ... ... ... ... ... ... ... ... ... ... ... ...  [420]
#FJ526438     G.. ... ... G.. ... ... ... ... ... ... ... ... ... ... ... ... ... ... ... ...  [420]
#FJ526437     ... ... ... ... ... ... ... ... ... ... ... ... ... ... ... ... ... ... ... ...  [420]
#AF417749     ... ... ... ... ... ... ... ... ... ... ... ... ... ... ... ... ... ... ... ...  [420]
#JX139608     ... ... ... ... ... ... ... ... ... ... ... ... ... ... ... ... ... ... ... ...  [420]

#PQ431427     TTT AGT AAC AGC TAC TGA TGT ATT ACA CTC ATG AAC TGT TCC CTC TTT AGG AGT AAA GGT  [480]
#PQ431428     ... ... ... ... ... ... ... ... ... ... ... ... ... ... ... ... ... ... ... ...  [480]
#PQ431429     ... ... ... ... ... ... ... ... ... ... ... ... ... ... ... ... ... ... ... ...  [480]
#PQ431430     ... ... ... ... ... ... ... ... ... ... ... ... ... ... ... ... ... ... ... ...  [480]
#PQ431431     ... ... ... ... ... ... ... ... ... ... ... ... ... ... ... ... ... ... ... ...  [480]
#MZ420723     ... ... ... ... ... ... ... ... ... ... ... ... ... ... ... ... ... ... ... ...  [480]
#MZ420724     ... ... ... ... ... ... ... ... ... ... ... ... ... ... ... ... ... ... ... ...  [480]
```

```
#MZ420725     ... ... ... ... ... ... ... ... ... ... ... ... ... ... ... ... ... ... ... ...   [480]
#MZ420726     ... ... ... ... ... ... ... ... ... ... ... ... ... ... ... ... ... ... ... ...   [480]
#MZ420727     ... ... ... ... ... ... ... ... ... ... ... ... ... ... ... ... ... ... ... ...   [480]
#MZ420728     ... ... ... ... ... ... ... ... ... ... ... ... ... ... ... ... ... ... ... ...   [480]
#MZ420729     ... ... ... ... ... ... ... ... ... ... ... ... ... ... ... ... ... ... ... ...   [480]
#MZ420730     ... ... ... ... ... ... ... ... ... ... ... ... ... ... ... ... ... ... ... ...   [480]
#MW431057     ... ... ... ... ... ... ... ... ... ... G.. ... ... ... ... ... ... ... ... ...   [480]
#KT899888     ... ... ... ... ... ... ... ... ... ... ... ... ... ... ... ... ... ... ... ...   [480]
#AF425844     ... ... ... ... ... ... ... ... ... ... ... ... ... ... ... ... ... ... ... ...   [480]
#DQ022847     ... ... ... ... ... ... ... ... ... ... ... ... ... ... ... ... ... ... ... ...   [480]
#DQ022846     ... ... ... ... ... ... ... ... ... ... ... ... ... ... ... ... ... ... ... ...   [480]
#DQ022845     ... ... ... ... ... ... ... ... ... ... ... ... ... ... ... ... ... ... ... ...   [480]
#DQ026675     ... ... ... ... ... ... ... ... ... ... ... ... ... ... ... ... ... ... ... ...   [480]
#AY883832     ... ... ... ... ... ... ... ... ... ... ... ... ... ... ... ... ... ... ... ...   [480]
#AY883830     ... ... ... ... ... ... ... ... ... ... ... ... ... ... ... ... ... ... ... ...   [480]
#AY883837     ... ... ... ... ... ... ... ... ... ... ... ... ... ... ... ... ... ... ... ...   [480]
#AY883836     ... ... ... ... ... ... ... ... ... ... ... ... ... ... ... ... ... ... ... ...   [480]
#AY883835     ... ... ... ... ... ... ... ... ... ... ... ... ... ... ... ... ... ... ... ...   [480]
#AY883834     ... ... ... ... ... ... ... ... ... ... ... ... ... ... ... ... ... ... ... ...   [480]
#AY883833     ... ... ... ... ... ... ... ... ... ... ... ... ... ... ... ... ... ... ... ...   [480]
#AY883831     ... ... ... ... ... ... ... ... ... ... ... ... ... ... ... ... ... ... ... ...   [480]
#KY863454     ... ... ... ... ... ... ... ... ... ... ... ... ... ... ... ... ... ... ... ...   [480]
#EF208912     ... ... ... ... ... ... ... ... ... ... ... ... ... ... ... ... ... ... ... ...   [480]
#FJ526438     ... ... ... ... ... ... ... ... ... ... ... ... ... ... ... ... ... ... ... ...   [480]
#FJ526437     ... ... ... ... ... ... ... ... ... ... ... ... ... ... ... ... ... ... ... ...   [480]
#AF417749     ... ... ... ... ... ... ... ... ... ... ... ... ... ... ... ... ... ... ... ...   [480]
#JX139608     ... ... ... ... ... ... ... ... ... ... ... ... ... ... ... ... ... ... ... ...   [480]

#PQ431427     AGA TGC TAC TCC TGG ACG ATT AAA TCA AAT TAA TTT CTT AAT TAA TCG ACC AGG ATT ATT   [540]
#PQ431428     ... ... ... ... ... ... ... ... ... ... ... ... ... ... ... ... ... ... ... ...   [540]
#PQ431429     ... ... ... ... ... ... ... ... ... ... ... ... ... ... ... ... ... ... ... ...   [540]
#PQ431430     ... ... ... ... ... ... ... ... ... ... ... ... ... ... ... ... ... ... ... ...   [540]
#PQ431431     ... ... ... ... ... ... ... ... ... ... ... ... ... ... ... ... ... ... ... ...   [540]
#MZ420723     ... ... ... ... ... ... ... ... ... ... ... ... ... ... ... ... ... ... ... ...   [540]
#MZ420724     ... ... ... ... ... ... ... ... ... ... ... ... ... ... ... ... ... ... ... ...   [540]
#MZ420725     ... ... ... ... ... ... ... ... ... ... ... ... ... ... ... ... ... ... ... ...   [540]
#MZ420726     ... ... ... ... ... ... ... ... ... ... ... ... ... ... ... ... ... ... ... ...   [540]
#MZ420727     ... ... ... ... ... ... ... ... ... ... ... ... ... ... ... ... ... ... ... ...   [540]
#MZ420728     ... ... ... ... ... ... ... ... ... ... ... ... ... ... ... ... ... ... ... ...   [540]
#MZ420729     ... ... ... ... ... ... ... ... ... ... ... ... ... ... A.. ... ... ... ... ...   [540]
#MZ420730     ... ... ... ... ... ... ... ... ... ... ... ... ... ... ... ... ... ... ... ...   [540]
#MW431057     ... ... ... ... ... ... ... ... ... ... ... ... ... ... ... ... ... ... ... ...   [540]
#KT899888     ... ... ... ... ... ... ... ... ... ... ... ... ... ... ... ... ... ... ... ...   [540]
#AF425844     ... ... ... ... ... ... ... ... ... ... ... ... ... ... ... ... ... ... ... ...   [540]
#DQ022847     ... ... ... ... ... ... ... ... ... ... ... ... ... ... ... ... ... ... ... ...   [540]
#DQ022846     ... ... ... ... ... ... ... ... ... ... ... ... ... ... ... ... ... ... ... ...   [540]
#DQ022845     ... ... ... ... ... ... ... ... ... ... ... ... ... ... ... ... ... ... ... ...   [540]
#DQ026675     ... ... ... ... ... ... ... ... ... ... ... ... ... ... ... ... ... ... ... ...   [540]
#AY883832     ... ... ... ... ... ... ... ... ... ... ... ... ... ... ... ... ... ... ... ...   [540]
#AY883830     ... ... ... ... ... ... ... ... ... ... ... ... ... ... ... ... ... ... ... ...   [540]
#AY883837     ... ... ... ... ... ... ... ... ... ... ... ... ... ... ... ... ... ... ... ...   [540]
#AY883836     ... ... ... ... ... ... ... ... ... ... ... ... ... ... ... ... ... ... ... ...   [540]
#AY883835     ... ... ... ... ... ... ... ... ... ... ... ... ... ... ... ... ... ... ... ...   [540]
#AY883834     ... ... ... ... ... ... ... ... ... ... ... ... ... ... ... ... ... ... ... ...   [540]
#AY883833     ... ... ... ... ... ... ... ... ... ... ... ... ... ... ... ... ... ... ... ...   [540]
#AY883831     ... ... ... ... ... ... ... ... ... ... ... ... ... ... ... ... ... ... ... ...   [540]
#KY863454     ... ... ... ... ... ... ... ... ... ... ... ... ... ... ... ... ... ... ... ...   [540]
#EF208912     ... ... ... ... ... ... ... ... ... ... ... ... ... ... ... ... ... ... ... ...   [540]
#FJ526438     ... ... ... ... ... ... ... ... ... ... ... ... ... ... ... ... ... ... ... ...   [540]
#FJ526437     ... ... ... ... ... ... ... ... ... ... ... ... ... ... ... ... ... ... ... ...   [540]
#AF417749     ... ... ... ... ... ... ... ... ... ... ... ... ... ... ... ... ... ... ... ...   [540]
#JX139608     ... ... ... ... ... ... ... ... ... ... ... ... ... ... ... ... ... ... ... ...   [540]
```

```
#PQ431427    TTT TGG TCA ATG TTC AGA AA [560]
#PQ431428    ... ... ... ... ... ... .. [560]
#PQ431429    ... ... ... ... ... ... .. [560]
#PQ431430    ... ... ... ... ... ... .. [560]
#PQ431431    ... ... ... ... ... ... .. [560]
#MZ420723    ... ... ... ... ... ... .. [560]
#MZ420724    ... ... ... ... ... ... .. [560]
#MZ420725    ... ... ... ... ... ... .. [560]
#MZ420726    ... ... ... ... ... ... .. [560]
#MZ420727    ... ... ... ... ... ... .. [560]
#MZ420728    ... ... ... ... ... ... .. [560]
#MZ420729    ... ... ... ... ... ... .. [560]
#MZ420730    ... ... ... ... ... ... .. [560]
#MW431057    ... ... ... ... ... ... .. [560]
#KT899888    ... ... A.. ... ... ... .. [560]
#AF425844    ... ... A.. ... ... ... .. [560]
#DQ022847    ... ... A.. ... ... ... .. [560]
#DQ022846    ... ... A.. ... ... ... .. [560]
#DQ022845    ... ... A.. ... ... ... .. [560]
#DQ026675    ... ... A.. ... ... ... .. [560]
#AY883832    ... ... A.. ... ... ... .. [560]
#AY883830    ... ... A.. ... ... ... .. [560]
#AY883837    ... ... A.. ... ... ... .. [560]
#AY883836    ... ... A.. ... ... ... .. [560]
#AY883835    ... ... A.. ... ... ... .. [560]
#AY883834    ... ... A.. ... ... ... .. [560]
#AY883833    ... ... A.. ... ... ... .. [560]
#AY883831    ... ... A.. ... ... ... .. [560]
#KY863454    ... ... A.. ... ... ... .. [560]
#EF208912    ... ... A.. ... ... ... .. [560]
#FJ526438    ... ... A.. ... ... ... .. [560]
#FJ526437    ... ... A.. ... ... ... .. [560]
#AF417749    ... ... A.. ... ... ... .. [560]
#JX139608    ... C.. A.. ... ... ... .. [560]
```
