# Supplementary material for: Phylogeographic Patterns and Genetic Diversity of Anopheles stephensi: Implications for Global Malaria Transmission
Source: Trop Med Infect Dis. 2025 Apr 16;10(4):109. doi: 10.3390/tropicalmed10040109 (PMC12031451; doi:10.3390/tropicalmed10040109)
Supplement: Supplementary file 1 [file tropicalmed-10-00109-s001.zip › Supplementary File S4 ITS2 Sequences.pdf]

```
# PQ423040               CAAGTTATCT CAACACTCTA AC-CAAACTG ACCGTCCTGA CCCCATCATA GGGGGGTAGG  [ 60]
# PQ423041               .......... .......... ..-....... .......... .......... ..........  [ 60]
# PQ423042               .......... .......... ..-....... .......... .......... ..........  [ 60]
#MZ269267                .......... .......... ..-....... .......... .......... ..........  [ 60]
#MZ269268                .......... .......... ..-....... .......... .......... ..........  [ 60]
#MZ269269                .......... .......... ..-....... .......... .......... ..........  [ 60]
#MZ269270                .......... .......... ..-....... .......... .......... ..........  [ 60]
#MZ269271                .......... .......... ..-....... .......... .......... ..........  [ 60]
#MW017363                .......... .......... ..-....... .......... .......... ..........  [ 60]
#MW017364                .......... .T.....C.. ..-....... .......... .......... ..........  [ 60]
#KM052589                .......... .......... ..-....... .......... .......... ..........  [ 60]
#KM052590                .......... .......... ..-....... .......... .......... ..........  [ 60]
#AY702486                .......... .......... ..-....... .......... .......... ..........  [ 60]
#AY702485                .......... .......... ..-....... .......... .......... ..........  [ 60]
#AY702484                .......... .......... ..-....... .......... .......... ..........  [ 60]
#AY702483                .......... .......... ..-....... .......... .......... ..........  [ 60]
#AY702482                .......... .......... ..-....... .......... .......... ..........  [ 60]
#AY365050                .......... .......... ..-....... .......... .......... ..........  [ 60]
#AY365049                .......... .......... ..-....... .......... .......... ..........  [ 60]
#AY702490                .......... .......... ..-....... .......... .......... ..........  [ 60]
#AY157316                .......... .......... ..-....... .......... .......... ..........  [ 60]
#AY157678                .......... .......... ..-....... .......... .......... ..........  [ 60]
#EU359681                .......... .......... ..-....... .......... .......... ..........  [ 60]
#EU359680                .......... .......... ..-....... .......... .......... ..........  [ 60]
#EU359679                .......... .......... ..-....... .......... .......... ..........  [ 60]
#EU359678                .......... .......... ..-....... .......... .......... ..........  [ 60]
#EU359677                .......... .......... ..-....... .......... .......... ..........  [ 60]
#EU359676                .......... .......... ..-....... .......... .......... ..........  [ 60]
#EU359675                .......... .......... ..-....... .......... .......... ..........  [ 60]
#EU359674                .......... .......... ..-....... .......... .......... ..........  [ 60]
#EU359673                .......... .......... ..-....... .......... .......... ..........  [ 60]
#EU359672                .......... .......... ..-....... .......... .......... ..........  [ 60]
#EU359671                .......... .......... ..-....... .......... .......... ..........  [ 60]
#EU359670                .......... .......... ..-....... .......... .......... ..........  [ 60]
#EU359669                .......... .......... ..-....... .......... .......... ..........  [ 60]
#EU359668                .......... .......... ..-....... .......... .......... ..........  [ 60]
#EU359667                .......... .......... ..-....... .......... .......... ..........  [ 60]
#EU359666                .......... .......... ..-....... .......... .......... ..........  [ 60]
#EU359665                .......... .......... ..-....... .......... .......... ..........  [ 60]
#EU359664                .......... .......... ..-....... .......... .......... ..........  [ 60]
#EU359663                .......... .......... ..-....... .......... .......... ..........  [ 60]
#EU359662                .......... .......... ..-....... .......... .......... ..........  [ 60]
#EU359661                .......... .......... ..-....... .......... .......... ..........  [ 60]
#EU346653                .......... .......... ..-....... .......... .......... ..........  [ 60]
#EU346652                .......... .......... ..-....... .......... .......... ..........  [ 60]
#DQ662409                .......... .......... ..-....... .......... .......... ..........  [ 60]
#HQ703001                .......... .......... ..-....... .......... .......... ..........  [ 60]
#AM231290.1An.gambiae    A--------- ---TT..TG. GTG.CT...A .TTAC.AAAG T.T...TTAG TTAACTACA.  [ 60]

#PQ423040                CTGTCGCAGC ATGGCGTGCT CGGATCCGCA TCCTTGTCGG GACCGTGGGC GCTGAAAGTG  [120]
#PQ423041                .......... .......... .......... .......... .......... ..........  [120]
#PQ423042                .......... .......... .......... .......... .......... ..........  [120]
#MZ269267                .......... .......... .......... .......... .......... ..........  [120]
#MZ269268                .......... .......... .......... .......... .......... ..........  [120]
#MZ269269                .......... .......... .......... .......... .......... ..........  [120]
#MZ269270                .......... .......... .......... .......... .......... ..........  [120]
```

```
#MZ269271                  .......... .......... .......... .......... .......... ..........  [120]
#MW017363                  .......... .......... .......... .......... .......... ..........  [120]
#MW017364                  .......... .......... .......... .......... .......... ..........  [120]
#KM052589                  .......... .......... .......... .......... .......... ..........  [120]
#KM052590                  .......... .......... .......... .......... .......... ..........  [120]
#AY702486                  .......... .......... .......... .......... .......... ..........  [120]
#AY702485                  .......... .......... .......... .......... .......... ..........  [120]
#AY702484                  .......... .......... .......... .......... .......... ..........  [120]
#AY702483                  .......... .......... .......... .......... .......... ..........  [120]
#AY702482                  .......... .......... .......... .......... .......... ..........  [120]
#AY365050                  .......... .......... .......... .......... .......... ..........  [120]
#AY365049                  .......... .......... .......... .......... .......... ..........  [120]
#AY702490                  .......... .......... .......... .......... .......... ..........  [120]
#AY157316                  .......... .......... .......... .......... .......... ..........  [120]
#AY157678                  .......... .......... .......... .......... .......... ..........  [120]
#EU359681                  .......... .......... .......... .......... .......... ..........  [120]
#EU359680                  .......... .......... .......... .......... .......... ..........  [120]
#EU359679                  .......... .......... .......... .......... .......... ..........  [120]
#EU359678                  .......... .......... .......... .......... .......... ..........  [120]
#EU359677                  .......... .......... .......... .......... .......... ..........  [120]
#EU359676                  .......... .......... .......... .......... .......... ..........  [120]
#EU359675                  .......... .......... .......... .......... .......... ..........  [120]
#EU359674                  .......... .......... .......... .......... .......... ..........  [120]
#EU359673                  .......... .......... .......... .......... .......... ..........  [120]
#EU359672                  .......... .......... .......... .......... .......... ..........  [120]
#EU359671                  .......... .......... .......... .......... .......... ..........  [120]
#EU359670                  .......... .......... .......... .......... .......... ..........  [120]
#EU359669                  .......... .......... .......... .......... .......... ..........  [120]
#EU359668                  .......... .......... .......... .......... .......... ..........  [120]
#EU359667                  .......... .......... .......... .......... .......... ..........  [120]
#EU359666                  .......... .......... .......... .......... .......... ..........  [120]
#EU359665                  .......... .......... .......... .......... .......... ..........  [120]
#EU359664                  .......... .......... .......... .......... .......... ..........  [120]
#EU359663                  .......... .......... .......... .......... .......... ..........  [120]
#EU359662                  .......... .......... .......... .......... .......... ..........  [120]
#EU359661                  .......... .......... .......... .......... .......... ..........  [120]
#EU346653                  .......... .......... .......... .......... .......... ..........  [120]
#EU346652                  .......... .......... .......... .......... .......... ..........  [120]
#DQ662409                  .......... .......... .......... .......... .......... ..........  [120]
#HQ703001                  .......... .......... .......... .......... .......... ..........  [120]
#AM231290.1An.gambiae      TG.C..TCCG CGAAG....C ...G..AT.C GA.GCACT.. .-.G..C.CT .TGC.T.A..  [120]

#PQ423040                  AGAGTGCTAA CACAGAGAGA CATACGAGGT ATGGTACAC- AAACCTAAAC ACACACACAC  [180]
#PQ423041                  .......... .......... ....T..... .........- .......... ..........  [180]
#PQ423042                  .......... .......... .......... .........- .......... ..........  [180]
#MZ269267                  .......... .......... .......... .........- .......... ..........  [180]
#MZ269268                  .......... .......... .......... .........- .......... ..........  [180]
#MZ269269                  .......... .......... .......... .........- .......... ..........  [180]
#MZ269270                  .......... .......... .......... .........- .......... ..........  [180]
#MZ269271                  .......... .......... .......... .........- .......... ..........  [180]
#MW017363                  G......... .......... .......... .........- .......... ..........  [180]
#MW017364                  .......... .......... .......... .........- .......... ..........  [180]
#KM052589                  .......... .......... .......... .........- .......... ..........  [180]
#KM052590                  .......... .......... .......... .........- .......... ..........  [180]
#AY702486                  .......... .......... .......... .........- .......... ..........  [180]
#AY702485                  .......... .......... .......... .........- .......... ..........  [180]
#AY702484                  .......... .......... .......... .........- .......... ..........  [180]
```

```
#AY702483                  .......... .......... .......... .........- .......... ..........  [180]
#AY702482                  .......... .......... .......... .........- .......... ..........  [180]
#AY365050                  .......... .......... .......... .........- .......... ..........  [180]
#AY365049                  .......... .......... .......... .........- .......... ..........  [180]
#AY702490                  .......... .......... .......... .........- .......... ..........  [180]
#AY157316                  .......... .......... .......... .........- .......... ..........  [180]
#AY157678                  .......... .......... .......... .........- .......... ..........  [180]
#EU359681                  .......... .......... .......... .........- .......... ..........  [180]
#EU359680                  .......... .......... .......... .........- .......... ..........  [180]
#EU359679                  .......... .......... .......... .........- .......... ..........  [180]
#EU359678                  .......... .......... .......... .........- .......... ..........  [180]
#EU359677                  .......... .......... .......... .........- .......... ..........  [180]
#EU359676                  .......... .......... .......... .........- .......... ..........  [180]
#EU359675                  .......... .......... .......... .........- .......... ..........  [180]
#EU359674                  .......... .......... .......... .........- .......... ..........  [180]
#EU359673                  .......... .......... .......... .........- .......... ..........  [180]
#EU359672                  .......... .......... .......... .........- .......... ..........  [180]
#EU359671                  .......... .......... .......... .........- .......... ..........  [180]
#EU359670                  .......... .......... .......... .........- .......... ..........  [180]
#EU359669                  .......... .......... .......... .........- .......... ..........  [180]
#EU359668                  .......... .......... .......... .........- .......... ..........  [180]
#EU359667                  .......... .......... .......... .........- .......... ..........  [180]
#EU359666                  .......... .......... .......... .........- .......... ..........  [180]
#EU359665                  .......... .......... .......... .........- .......... ..........  [180]
#EU359664                  .......... .......... .......... .........- .......... ..........  [180]
#EU359663                  .......... .......... .......... .........- .......... ..........  [180]
#EU359662                  .......... .......... .......... .........- .......... ..........  [180]
#EU359661                  .......... .......... .......... .........- .......... ..........  [180]
#EU346653                  .......... .......... .......... .........- .......... ..........  [180]
#EU346652                  .......... .......... .......... .........- .......... ..........  [180]
#DQ662409                  .......... .......... .......... .........- .......... ..........  [180]
#HQ703001                  .......... .......... .......... .........- .......... ..........  [180]
#AM231290.1An.gambiae      .C-.....TG GT.CCC.--- TC.G..G.TC C.C.GG.GTT G..AG.GG.. ..T.T.GAG.  [180]

#PQ423040                  ACATGTGAGC ATGG-GTGAA GAGAGAGCGC GCGTCAAGTC GCACGGTTCG ACCTCTAGTA  [240]
#PQ423041                  .......... ....-..... ...C...... .......... .......... ..........  [240]
#PQ423042                  .......... ....-..... ...C...... .......... .......... ..........  [240]
#MZ269267                  .......... ....-..... ...C...... .......... .......... ..........  [240]
#MZ269268                  .......... ....-..... ...C...... .......... .......... ..........  [240]
#MZ269269                  .......... ....-..... ...C...... .......... .......... ..........  [240]
#MZ269270                  .......... ....-..... ...C...... .......... .......... ..........  [240]
#MZ269271                  .......... ....-..... ...C...... .......... .......... ..........  [240]
#MW017363                  .......... ....-..... ...C...... .......... .......... ..........  [240]
#MW017364                  .......... ....-..... ...C...... .......... .......... ...T......  [240]
#KM052589                  .......... ....-..... ...C...... .......... .......... ..........  [240]
#KM052590                  .......... ....-..... ...C...... .......... .......... ..........  [240]
#AY702486                  .......... ....-..... ...C...... .......... .......... ..........  [240]
#AY702485                  .......... ....A..... ..AC...... .......... .......... ..........  [240]
#AY702484                  .......... ....-..... ...C...... .......... .......... ..........  [240]
#AY702483                  .......... ....-..... ...C...... .......... .......... ..........  [240]
#AY702482                  .......... ....-..... ...C...... .......... .......... ..........  [240]
#AY365050                  .......... ....-..... ...C...... .......... .......... ..........  [240]
#AY365049                  .......... ....-..... ...C...... .......... .......... ..........  [240]
#AY702490                  .......... ....-..... ...C...... .......... .......... ..........  [240]
#AY157316                  .......... ....-..... ...C...... .......... .......... ..........  [240]
#AY157678                  .......... ....-..... ...C...... .......... .......... ..........  [240]
#EU359681                  .......... ....-..... ...C...... .......... .......... ..........  [240]
```

```
#EU359680                 .......... ....-..... ...C...... .......... .......... ..........  [240]
#EU359679                 .......... ....-..... ...C...... .......... .......... ..........  [240]
#EU359678                 .......... ....-..... ...C...... .......... .......... ..........  [240]
#EU359677                 .......... ....-..... ...C...... .......... .......... ..........  [240]
#EU359676                 .......... ....-..... ...C...... .......... .......... ..........  [240]
#EU359675                 .......... ....-..... ...C...... .......... .......... ..........  [240]
#EU359674                 .......... ....-..... ...C...... .......... .......... ..........  [240]
#EU359673                 .......... ....-..... ...C...... .......... .......... ..........  [240]
#EU359672                 .......... ....-..... ...C...... .......... .......... ..........  [240]
#EU359671                 .......... ....-..... ...C...... .......... .......... ..........  [240]
#EU359670                 .......... ....-..... ...C...... .......... .......... ..........  [240]
#EU359669                 .......... ....-..... ...C...... .......... .......... ..........  [240]
#EU359668                 .......... ....-..... ...C...... .......... .......... ..........  [240]
#EU359667                 .......... ....-..... ...C...... .......... .......... ..........  [240]
#EU359666                 .......... ....-..... ...C...... .......... .......... ..........  [240]
#EU359665                 .......... ....-..... ...C...... .......... .......... ..........  [240]
#EU359664                 .......... ....-..... ...C...... .......... .......... ..........  [240]
#EU359663                 .......... ....-..... ...C...... .......... .......... ..........  [240]
#EU359662                 .......... ....-..... ...C...... .......... .......... ..........  [240]
#EU359661                 .......... ....-..... ...C...... .......... .......... ..........  [240]
#EU346653                 .......... ....-..... ...C...... .......... .......... ..........  [240]
#EU346652                 .......... ....-..... ...C...... .......... .......... ..........  [240]
#DQ662409                 .......... ....-..... ...C...... .......... .......... ..........  [240]
#HQ703001                 .TG......T G...-.A... .......... .......... A..G..C.AC .......A..  [240]
#AM231290.1An.gambiae     GT....TG.- ...C-..TTC .T.TTG.T.G TGT.TG.TG. .T.G..C.T. TGG.G.-..G  [240]

#PQ423040                 TCAACCAACG GATGTATCCA CCACAGCATA TAAGGTTATC ACCAATTGCA CGGGGACTTC  [300]
#PQ423041                 .......... .......... .......... .......... .......... ..........  [300]
#PQ423042                 .......... .......... .......... .......... .......... ..........  [300]
#MZ269267                 .......... .......... .......... .......... .......... ..........  [300]
#MZ269268                 .......... .......... .......... .......... .......... ..........  [300]
#MZ269269                 .......... .......... .......... .......... .......... ..........  [300]
#MZ269270                 .......... .......... .......... .......... .......... ..........  [300]
#MZ269271                 .......... .......... .......... .......... .......... ..........  [300]
#MW017363                 .......... .......... .......... .......... .......... ..........  [300]
#MW017364                 .......... .......... .......... .......... .......... ..........  [300]
#KM052589                 .......... .......... .......... .......... .......... ..........  [300]
#KM052590                 .......... .......... .......... .......... .......... ..........  [300]
#AY702486                 .......... .......... .......... .......... .......... ..........  [300]
#AY702485                 .......... .......... .......... .......... .......... ..........  [300]
#AY702484                 .......... .......... .......... .......... .......... ..........  [300]
#AY702483                 .......... .......... .......... .......... .......... ..........  [300]
#AY702482                 .......... .......... .......... .......... .......... ..........  [300]
#AY365050                 .......... .......... .......... .......... .......... ..........  [300]
#AY365049                 .......... .......... .......... .......... .......... ..........  [300]
#AY702490                 .......... .......... .......... .......... .......... ..........  [300]
#AY157316                 .......... .......... .......... .......... .......... ..........  [300]
#AY157678                 .......... .......... .......... .......... .......... ..........  [300]
#EU359681                 .......... .......... .......... .......... .......... ..........  [300]
#EU359680                 .......... .......... .......... .......... .......... ..........  [300]
#EU359679                 .......... .......... .......... .......... .......... ..........  [300]
#EU359678                 .......... .......... .......... .......... .......... ..........  [300]
#EU359677                 .......... .......... .......... .......... .......... ..........  [300]
#EU359676                 .......... .......... .......... .......... .......... ..........  [300]
#EU359675                 .......... .......... .......... .......... .......... ..........  [300]
#EU359674                 .......... .......... .......... .......... .......... ..........  [300]
#EU359673                 .......... .......... .......... .......... .......... ..........  [300]
```

```
#EU359672                 .......... .......... .......... .......... .......... ..........  [300]
#EU359671                 .......... .......... .......... .......... .......... ..........  [300]
#EU359670                 .......... .......... .......... .......... .......... ..........  [300]
#EU359669                 .......... .......... .......... .......... .......... ..........  [300]
#EU359668                 .......... .......... .......... .......... .......... ..........  [300]
#EU359667                 .......... .......... .......... .......... .......... ..........  [300]
#EU359666                 .......... .......... .......... .......... .......... ..........  [300]
#EU359665                 .......... .......... .......... .......... .......... ..........  [300]
#EU359664                 .......... .......... .......... .......... .......... ..........  [300]
#EU359663                 .......... .......... .......... .......... .......... ..........  [300]
#EU359662                 .......... .......... .......... .......... .......... ..........  [300]
#EU359661                 .......... .......... .......... .......... .......... ..........  [300]
#EU346653                 .......... .......... .......... .......... .......... ..........  [300]
#EU346652                 .......... .......... .......... .......... .......... ..........  [300]
#DQ662409                 .......... .......... .......... .......... .......... ..........  [300]
#HQ703001                 ..CC..C.GA T........C ....AT.... ..G..A.... ...TTG...G G..AC.....  [300]
#AM231290.1An.gambiae     ....G.--.. C...GT..G. A.TA.TGC.. CGTC...CC. GATGGCCA.C G.CA.T..A.  [300]

#PQ423040                 CACCGGTTGG CTCGGGTCGA GTAACACTTG CGGCCCAACG CGCTCGTATC TTTCCTCGCA  [360]
#PQ423041                 .......... .......... .......... .......... .......... ..........  [360]
#PQ423042                 .......... .......... .......... .......... .......... ..........  [360]
#MZ269267                 .......... .......... .......... .......... .......... ..........  [360]
#MZ269268                 .......... .......... .......... .......... .......... ..........  [360]
#MZ269269                 .......... .......... .......... .......... .......... ..........  [360]
#MZ269270                 .......... .......... .......... .......... .......... ..........  [360]
#MZ269271                 .......... .......... .......... .......... .......... ..........  [360]
#MW017363                 .......... .......... .......... .......... .......... ..........  [360]
#MW017364                 .......... .......... .......... .......... .......... ..........  [360]
#KM052589                 .......... .......... .......... .......... .......... ..........  [360]
#KM052590                 .......... .......... .......... .......... .......... ..........  [360]
#AY702486                 .......... .......... .......... .......... .......... ..........  [360]
#AY702485                 .......... .......... .......... .......... .......... ..........  [360]
#AY702484                 .......... .......... .......... .......... .......... ..........  [360]
#AY702483                 .......... .......... .......... .......... .......... ..........  [360]
#AY702482                 .......... .......... .......... .......... .......... ..........  [360]
#AY365050                 .......... .......... .......... .......... .......... ..........  [360]
#AY365049                 .......... .......... .......... .......... .......... ..........  [360]
#AY702490                 .......... .......... .......... .......... .......... ..........  [360]
#AY157316                 .......... .......... .......... .......... .......... ..........  [360]
#AY157678                 .......... .......... .......... .......... .......... ..........  [360]
#EU359681                 .......... .......... .......... .......... .......... ..........  [360]
#EU359680                 .......... .......... .......... .......... .......... ..........  [360]
#EU359679                 .......... .......... .......... .......... .......... ..........  [360]
#EU359678                 .......... .......... .......... .......... .......... ..........  [360]
#EU359677                 .......... .......... .......... .......... .......... ..........  [360]
#EU359676                 .......... .......... .......... .......... .......... ..........  [360]
#EU359675                 .......... .......... .......... .......... .......... ..........  [360]
#EU359674                 .......... .......... .......... .......... .......... ..........  [360]
#EU359673                 .......... .......... .......... .......... .......... ..........  [360]
#EU359672                 .......... .......... .......... .......... .......... ..........  [360]
#EU359671                 .......... .......... .......... .......... .......... ..........  [360]
#EU359670                 .......... .......... .......... .......... .......... ..........  [360]
#EU359669                 .......... .......... .......... .......... .......... ..........  [360]
#EU359668                 .......... .......... .......... .......... .......... ..........  [360]
#EU359667                 .......... .......... .......... .......... .......... ..........  [360]
#EU359666                 .......... .......... .......... .......... .......... ..........  [360]
#EU359665                 .......... .......... .......... .......... .......... ..........  [360]
```

```
#EU359664                    .......... .......... .......... .......... .......... ..........  [360]
#EU359663                    .......... .......... .......... .......... .......... ..........  [360]
#EU359662                    .......... .......... .......... .......... .......... ..........  [360]
#EU359661                    .......... .......... .......... .......... .......... ..........  [360]
#EU346653                    .......... .......... .......... .......... .......... ..........  [360]
#EU346652                    .......... .......... .......... .......... .......... ..........  [360]
#DQ662409                    .......... .......... .......... .......... .......... ..........  [360]
#HQ703001                    .C....G..T .......A.. .A....TG.. .......... .T...A...T .......AT.  [360]
#AM231290.1An.gambiae        TCT.CA--.. ..AAA....G C.CGT-..A. G.ATT.GGAA A...AAG--- --..GCT.T.  [360]

#PQ423040                    TCCAGTTGCA GTCGAGAGAC GTGCTCACGC CGTGTGGGTG AATGAGGTTA GCACGA-CAG  [420]
#PQ423041                    .......... ....C..... .......... .......... .G........ ......-...  [420]
#PQ423042                    .......... ....C..... .......... .......... .G........ ......-...  [420]
#MZ269267                    .......... ....C..... .......... .......... .G........ ......-...  [420]
#MZ269268                    .......... ....C..... .......... .......... .G........ ......-...  [420]
#MZ269269                    .......... ....C..... .......... .......... .G........ ......-...  [420]
#MZ269270                    .......... ....C..... .......... .......... .G........ ......-...  [420]
#MZ269271                    .......... ....C..... .......... .......... .G........ ......-...  [420]
#MW017363                    .......... ....C..... .......... .......... .......... ......-...  [420]
#MW017364                    .......... ....C..... .......... ......A... .G........ ......-...  [420]
#KM052589                    .......... ....C..... .......... .......... .G........ ......-...  [420]
#KM052590                    .......... ....C..... .......... .......... .G........ ......-...  [420]
#AY702486                    .......... ....C..... .......... .......... .G........ ......-...  [420]
#AY702485                    .......... ....C..... .......... .......... .G........ ......-...  [420]
#AY702484                    .......... ....C..... .......... .......... .G........ ......-...  [420]
#AY702483                    .......... ....C..... .......... .......... .G........ ......A...  [420]
#AY702482                    .......... ....C..... .......... .......... .G........ ......-...  [420]
#AY365050                    .......... ....C..... .......... .......... .G........ ......-...  [420]
#AY365049                    .......... ....C..... .......... .......... .G........ ......-...  [420]
#AY702490                    .......... ....C..... .......... .......... .G........ ......-...  [420]
#AY157316                    .......... ....C..... .......... .......... .G........ ......-...  [420]
#AY157678                    .......... ....C..... .......... .......... .G........ ......-...  [420]
#EU359681                    .......... ....C..... .......... .......... .G........ ......-...  [420]
#EU359680                    .......... ....C..... .......... .......... .G........ ......-...  [420]
#EU359679                    .......... ....C..... .......... .......... .G........ ......-...  [420]
#EU359678                    .......... ....C..... .......... .......... .G........ ......-...  [420]
#EU359677                    .......... ....C..... .......... .......... .G........ ......-...  [420]
#EU359676                    .......... ....C..... .......... .......... .G........ ......-...  [420]
#EU359675                    .......... ....C..... .......... .......... .G........ ......-...  [420]
#EU359674                    .......... ....C..... .......... .......... .G........ ......-...  [420]
#EU359673                    .......... ....C..... .......... .......... .G........ ......-...  [420]
#EU359672                    .......... ....C..... .......... .......... .G........ ......-...  [420]
#EU359671                    .......... ....C..... .......... .......... .G........ ......-...  [420]
#EU359670                    .......... ....C..... .......... .......... .G........ ......-...  [420]
#EU359669                    .......... ....C..... .......... .......... .G........ ......-...  [420]
#EU359668                    .......... ....C..... .......... .......... .G........ ......-...  [420]
#EU359667                    .......... ....C..... .......... .......... .G........ ......-...  [420]
#EU359666                    .......... ....C..... .......... .......... .G........ ......-...  [420]
#EU359665                    .......... ....C..... .......... .......... .G........ ......-...  [420]
#EU359664                    .......... ....C..... .......... .......... .G........ ......-...  [420]
#EU359663                    .......... ....C..... .......... .......... .G........ ......-...  [420]
#EU359662                    .......... ....C..... .......... .......... .G........ ......-...  [420]
#EU359661                    .......... ....C..... .......... .......... .G........ ......-...  [420]
#EU346653                    .......... ....C..... .......... .......... .G........ C.....-...  [420]
#EU346652                    .......... ....C..... .......... .......... .G........ ......-...  [420]
#DQ662409                    .......... .A..C..... .......... .......... .G........ ......-...  [420]
#HQ703001                    ...TTG.... C........T .......... ......T.A. .GA..T.A.. A..AC.-...  [420]
```

```
#AM231290.1An.gambiae    A.TCA.GTGG .C.C.T.C.. .GCG.TG... TACCAC.C.A .G.T..CCCT A..TATA..A  [420]

#PQ423040                GGGTGATTTA TCACCGCTTC TCCCGTCGCA T-CATTGTGA CAGTG-GAGT CT [472]
#PQ423041                .......... .......... .......... .-........ .....-.... .. [472]
#PQ423042                .......... .......... .......... .-........ .....-.... .. [472]
#MZ269267                .......... .......... .......... .-........ .....-.... .. [472]
#MZ269268                .......... .......... .......... .-........ .....-.... .. [472]
#MZ269269                .......... .......... .......... .-........ .....-.... .. [472]
#MZ269270                .......... .......... .......... .-........ .....-.... .. [472]
#MZ269271                .......... .......... .......... .-........ .....-.... .. [472]
#MW017363                .......... .......... .......... .-........ .....-.... .. [472]
#MW017364                .......... .......... .......... .-........ .....-.... .. [472]
#KM052589                .......... .......... .......... .-........ .....-.... .. [472]
#KM052590                .......... .......... .......... .-........ .....-.... .. [472]
#AY702486                .......... .......... .......... .-........ .....-.... .. [472]
#AY702485                .......... .......... .......... .A........ .....-.... .. [472]
#AY702484                .......... .......... .......... .-........ .....-.... .. [472]
#AY702483                .......... .......... .......... .-........ .....-.... .. [472]
#AY702482                .......... .......... .......A.. .-........ .....-.... .. [472]
#AY365050                .......... .......... .......... .-........ .....-.... .. [472]
#AY365049                .......... .......... .......... .-........ .....-.... .. [472]
#AY702490                .......... .......... .......... .-........ .....-.... .. [472]
#AY157316                .......... .......... .......... .-........ .....-.... .. [472]
#AY157678                .......... .......... .......... .-........ .....-.... .. [472]
#EU359681                .......... .......... .......... .-........ .....-.... .. [472]
#EU359680                .......... .......... .......... .-........ .....-.... .. [472]
#EU359679                .......... .......... .......... .-........ .....-.... .. [472]
#EU359678                .......... .......... .......... .-........ .....-.... .. [472]
#EU359677                .......... .......... .......... .-........ .....-.... .. [472]
#EU359676                .......... .......... .......... .-........ .....-.... .. [472]
#EU359675                .......... .......... .......... .-........ .....-.... .. [472]
#EU359674                .......... .......... .......... .-........ .....-.... .. [472]
#EU359673                .......... .......... .......... .-........ .....-.... .. [472]
#EU359672                .......... .......... .......... .-........ .....-.... .. [472]
#EU359671                .......... .......... .......... .-........ .....-.... .. [472]
#EU359670                .......... .......... .......... .-........ .....-.... .. [472]
#EU359669                .......... .......... .......... .-........ .....-.... .. [472]
#EU359668                .......... .......... .......... .-........ .....-.... .. [472]
#EU359667                .......... .......... .......... .-........ .....-.... .. [472]
#EU359666                .......... .......... .......... .-........ .....-.... .. [472]
#EU359665                .......... .......... .......... .-........ .....-.... .. [472]
#EU359664                .......... .......... .......... .-........ .....-.... .. [472]
#EU359663                .......... .......... .......... .-........ .....-.... .. [472]
#EU359662                .......... .......... .......... .-........ .....-.... .. [472]
#EU359661                .......... .......... .......... .-........ .....-.... .. [472]
#EU346653                .......... .......... .......... .-........ .....-.... .. [472]
#EU346652                .......... .......... .......... .-........ .....A.... .. [472]
#DQ662409                .......... .......... .......... .-........ .....-.... .. [472]
#HQ703001                .T.A...A.. ...G...C.. ..GTC..A.. .-.TG..A.. ...GA-..C. .. [472]
#AM231290.1An.gambiae    .CA.C.ACCC A.GG.A.GGG CGTA.CT.T. ATAC..AC.T .TCG.----- -- [472]
```
